# Engaging Older Adults and Staff in the Co-Design and Evaluation of Socially Assistive Robot and Virtual Reality Activities for Long-Term Care: User-Centered Study

**DOI:** 10.2196/75288

**Published:** 2025-12-02

**Authors:** Ritam Ghosh, Nibraas Khan, Miroslava Migovich, Judith A Tate, Cathy A Maxwell, Paul A Newhouse, Douglas W Scharre, Alai Tan, Lorraine C Mion, Nilanjan Sarkar

**Affiliations:** 1 Department of Electrical and Computer Engineering School of Engineering Vanderbilt University Nashville, TN United States; 2 Department of Computer Science School of Engineering Vanderbilt University Nashville, TN United States; 3 Department of Mechanical Engineering School of Engineering Vanderbilt University Nashville, TN United States; 4 College of Nursing The Ohio State University Columbus, OH United States; 5 College of Nursing University of Utah Salt Lake City, UT United States; 6 Center for Cognitive Medicine Department of Psychiatry and Behavioral Sciences Vanderbilt University Medical Center Nashville, TN United States; 7 Department of Neurology The Ohio State University Wexner Medical Center Columbus, OH United States

**Keywords:** apathy, long-term care settings, nonimmersive virtual reality, older adults, socially assistive robots

## Abstract

**Background:**

Apathy is common among older adults residing in long-term care (LTC) and impairs quality of life for both older adults and care providers. Few pharmacological remedies exist, and nonpharmacologic approaches that engage those with apathy require extensive personnel time. Thus, technological approaches have been encouraged, including virtual reality (VR) and socially assistive robots (SAR). Despite a growing interest in their use, input from older adults and staff is often absent in their design. Involving older adults in the development of interactive health technologies is necessary to enhance the functionality, usability, and likelihood of promoting the intended health outcomes.

**Objective:**

We aimed to design and evaluate SAR and nonimmersive VR (SAR-VR) activities for pairs of older adults that would encourage human-to-human interaction, an essential activity to mitigate apathy.

**Methods:**

We implemented a multistep, user-centered design. A humanoid and dog SAR were used in combination with nonimmersive VR activities for pairs of older adults. An interdisciplinary team of engineers, nurses, and physicians collaborated with older adults and staff to create 4 activity prototypes, 3 with the humanoid robot and 1 with the dog robot. A total of 14 older adults at 2 sites participated in the design and evaluation of the different components of the system throughout all stages. Site 1 participants were instrumental in the development, and Site 2 participants validated the prototype activities. Data were collected at each session via observations, interviews, and a 6-item questionnaire that rated their degree of comfort and confidence in (1) using the wands, (2) interacting with the robot, and (3) interacting with the nonimmersive VR environment using a 5-point Likert response. Additionally, 5 staff from Site 2 were recruited to evaluate the ease of setting up and running the system at 2 different sessions. After each session, the system setup and interface were refined based on their feedback.

**Results:**

A total of 4 of 6 older adults (mean age 85, SD 9.3 years; 2 male) at Site 1 completed field testing development, and 8 residents (mean age 80, SD 4.7 years; 2 male) at Site 2 completed field testing validation. Participant comfort and confidence increased significantly over successive iterations of the system across most categories (Site 1: Wilcoxon signed rank test *P*=.03; Site 2: Wilcoxon signed rank test *P*<.001). Additionally, 5 LTC staff members successfully set up the system with minimal cueing from the researchers, demonstrating the usability of the system for caregivers. Iterative design changes incorporated hardware, software, and activity domains.

**Conclusions:**

These initial results demonstrate that LTC older adults and staff are capable and critical to the development and implementation of SAR-VR activities. Future studies are needed to evaluate the feasibility of implementation and effectiveness in reducing apathy.

**Trial Registration:**

ClinicalTrials.gov NCT05178992; https://clinicaltrials.gov/study/NCT05178992

## Introduction

In the United States, 1.3 million older adults reside in nursing homes, and another 918,000 reside in assisted living facilities [1], representing 3.8% of the older adult population. However, the lifetime probability of requiring long-term care (LTC) is 70% for those who survive to the age of 65 years, and 15% will spend 2 or more years in a nursing home [2]. The presence of apathy complicates the care delivery to these vulnerable older adults. Apathy is a condition that results in a lack of initiative, loss of interest in daily activities, and reluctance toward social interactions [3]. Apathy is common among LTC residents, occurring in up to 82% of those with dementia and up to a third of those with depression [4-6]. Older adults experiencing apathy often remain disengaged with their surroundings and may require significant prompting and encouragement to perform basic activities, such as speaking, eating, bathing, or walking. Apathy has significant deleterious consequences, including accelerated cognitive decline, further functional deficits, diminished quality of life, and increased rates of mortality [7]. Not only does apathy negatively impact the older adult, but it also causes significant burden and stress on family and staff [3,4].

Despite the scope and significance of apathy, few pharmacologic options are available [8]; thus, managing apathy requires effective nonpharmacologic strategies. Activities that combine motor-based and cognitive aspects with social aspects, that is, human-to-human interaction (HHI), appear to be the most effective for enhancing engagement and reducing apathy among older adults [9-13]*.* Unfortunately, designing and delivering these multimodal activities require significant personnel resources, and many LTCs have inadequate staffing, either in labor quantity or skill [14,15]. Thus, technology has been suggested to complement existing staff and facilitate the delivery of care to older adults [16-18]. Although most technological interventions in LTCs have focused on the electronic health record, telehealth, and wearables, there is a growing interest in the use of nonimmersive or immersive virtual reality (VR) [19-21] or socially assistive robots (SARs) [22,23] to enhance function or quality of life. Our work focuses on the combination of SAR with nonimmersive VR, hereafter referred to as SAR-VR.

Both SARs and VR have shown promising results for older adults [19,22,24], and each has its strengths as well as limitations that a combined approach can address. SARs have the distinct advantage of physical presence, the ability to emulate body language, and use of nonverbal cues that foster trust and engagement beyond what on-screen avatars typically achieve [25,26]. However, the readily available commercial SARs have limited capability to manipulate physical objects, while custom SARs are expensive and often designed for a specific application, limiting their activities. Conversely, nonimmersive VRs allow for the emulation of various physical scenarios displayed on a standard computer monitor that can provide a greater range of activities compared to those provided by robots alone. VR activities can also be easily adjusted to accommodate an individual’s physical capabilities (eg, impaired mobility, reduced strength, diminished range of motion). However, nonimmersive VR limits the interaction to the use of a mouse, joystick, or remote control, which may be difficult for older adults with reduced motor dexterity and proprioception in hands and wrists [27-29].

An SAR-VR system leverages the strengths of both modalities while compensating for their weaknesses. The SAR provides embodied, socially engaging interaction that motivates participation, while the VR component expands the scope and adaptability of activities beyond the robot’s physical affordances. Together, the two create more holistic activities that are simultaneously socially engaging, physically accessible, and adaptable to the needs of aging populations. Recent work has demonstrated the successful combination of assistive robotics with nonimmersive VR for other populations, including stroke rehabilitation [30,31], rehabilitation for children with motor skill impairment [32], and conducting video lectures [33]. In our earlier pilot work [34], we successfully combined an SAR with a nonimmersive VR-based activity using gesture control for engaging pairs of older adults residing in the community and subsequently in LTCs. The activity was designed based on input from geriatric experts and refined based on older adults’ usability testing.

Our long-term goal is to use SAR-VR systems to address apathy among older LTC residents. As an initial step, we needed to create SAR-VR activities that combined cognitive, physical, and social aspects and encouraged HHI during the activities. Given the range of physical and cognitive impairments in this population, we deemed it essential to involve older LTC adults in the development of these activities to enhance the functionality, usability, and likelihood of promoting their use [35,36]. However, older LTC adults are often absent during the design of interactive health technologies [37]. To enhance the likelihood that the designed SAR-VR activities could effectively engage pairs of older LTC adults with varying levels of physical and cognitive function, we used a user-centered design (UCD) approach.

UCD, variously termed as human-centered design or participatory design, is a fundamental engineering philosophy in the design and development of products. It is a structured, iterative method that focuses on the end user throughout the entire design and development [38], but little information is available about the practical implementation of UCD methods with older adults with cognitive impairment [39]. Challenges to UCD implementation with this population include not only the characteristics of the older adults but also organizational and staff characteristics.

Our aim was to design and evaluate SAR-VR activities for pairs of older adults that would encourage HHI, an essential activity to mitigate apathy. To address gaps in knowledge and facilitate future work involving SAR-VR with this vulnerable population, we present our experiences in conducting UCD with older adults with cognitive impairment and characterize the challenges encountered in developing the SAR-VR system and paired activities.

## Methods

### User-Centered Study Design of the SAR-VR System

**Figure 1:**
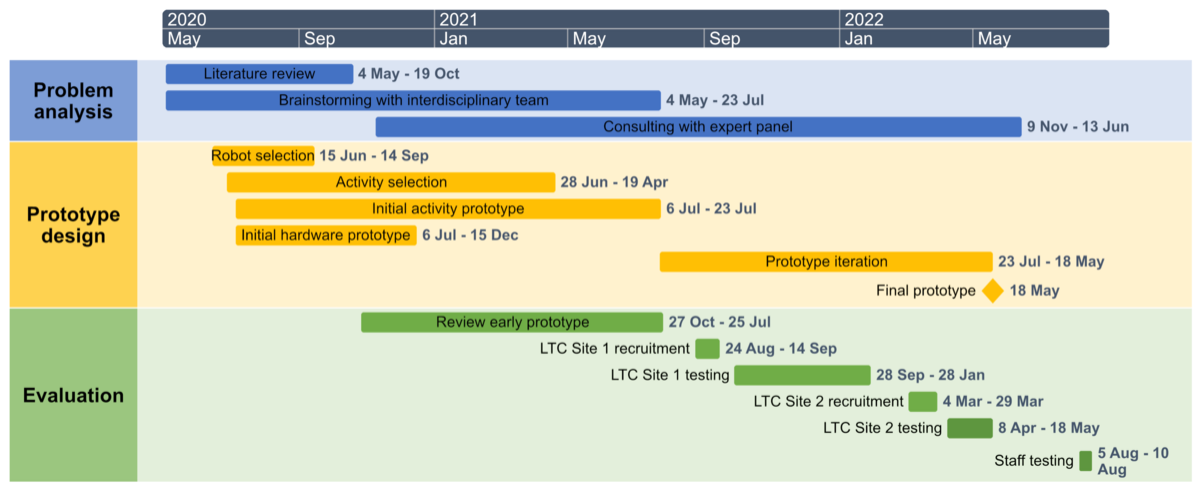
Timeline for user-centered study process. LTC: long-term care.

We implemented a multistep UCD process to design and evaluate SAR-VR activities that would encourage HHI mediated through human-computer interaction (HCI; nonimmersive VR) and human-robot interaction (HRI). Three overarching UCD principles guided the process throughout: (1) focus on users and tasks, (2) measure usability empirically, and (3) design and test usability iteratively [40,41]. Figure 1 displays the UCD timeline. There were distinct phases and activities to the UCD study process that are detailed below. These included problem analysis, prototype design development and evaluation, and field testing and validation.

### Participant Recruitment

Participants consisted of older adults residing in an assisted living facility and staff. Eligibility criteria for older adults were age 65 years and older, at least 3 months of residence at the facility, the ability to hear, speak, and understand English, and the ability to sit comfortably and move both arms. Staff criteria included involvement directly or indirectly with the activities offered to residents within the setting and willingness to work with the researchers on the SAR-VR system. Investigators contacted several assisted living facilities by phone and email. Facilities needed to be within an hour’s drive of the Vanderbilt University School of Engineering. Using institutional review board (IRB)–approved script, we explained the study purpose and our needs to conduct the study, that is, physical space adequate for the robot and nonimmersive VR setup, physical space conducive to the activity and evaluation (eg, adequate lighting and minimal noise or interruptions), and willingness to allow staff to interact with the research staff. Upon receiving permission from site administrators, Vanderbilt University engineers spoke to potentially interested residents at a resident meeting and to staff at a staff meeting, demonstrated the robot, and provided information regarding the study needs. Interested residents and staff contacted the research team directly, and a meeting was arranged to determine eligibility and conduct the informed consent process.

### Phase 1: Problem Analysis

The first step was problem analysis, that is, to specify the context of use of SAR-VR activities with pairs of frail older adults. First, our interdisciplinary team brought expertise to the area of inquiry, including engineers from various disciplines (ie, mechanical engineering, electrical engineering, and computer science) as well as nurses and physicians who specialized in geriatrics and LTC. Based on our areas of expertise, we searched PubMed to (1) identify aspects of activities that effectively engage older LTC adults, especially those who have apathy, (2) SAR characteristics acceptable to older adults, and (3) nonimmersive VR factors suitable to older adults.

Results from our literature review were discussed with an interdisciplinary advisory panel (n=8), consisting of LTC activity directors, nurses, managers, and a doctoral-prepared occupational therapist who specialized in geriatrics. Based on the literature review and conversations with the advisory panel and older adults, inspiration for activities was based on real-life activities the older adults performed at their LTCs and hobbies they enjoyed. Criteria for activities included (1) flexibility to accommodate older adults with varying levels of physical and cognitive abilities, (2) varying levels of collaboration required between 2 older adults, and (3) sufficient variation of activities to minimize monotony. Lastly, the system must have a user-friendly graphical user interface so that LTC staff can easily operate it and older adults can manipulate it. We conducted multiple design iterations and internal testing of prototype activities in the Vanderbilt University Engineering Laboratory. We presented ongoing work over several meetings with the advisory panel. The 2 LTC residents reviewed and approved the initial prototype activities for field testing via teleconferencing (due to COVID-19 isolation protocols).

### Phase 2: Early Prototype Design

This phase focused on the choice of robots, software, and hardware development, and the choice of activities for each robot type.

#### Choice of Robots

In our earlier work [34], we found that older adults varied in their responsiveness and enjoyment to 2 types of SARs, humanoid and animal. For this study, we used NAO from SoftBank Robotics as our humanoid robot [42]. NAO’s plastic body is child-sized at 58 cm and has 25 degrees of freedom with sensors ranging from tactile to audio microphone arrays. It has been used in numerous clinical and research settings, given its ease of use, sufficient expressive power for tasks, and its open architecture, allowing custom intelligent software development. We chose NAO because its limitations are not detrimental to our goals, its open architecture is capable of our specific modifications toward closed-loop intelligent interaction, and it is not prohibitively expensive. In our prior work, we augmented NAO’s vision with a Kinect for Windows sensor (Microsoft Corp) for gesture recognition and gaze estimation using our prior developed algorithms [34,43]. We also designed a rule-based finite state machine method to recognize physical gestures (Multimedia Appendix 1).

We chose Aibo from SONY [44] as our dog robot. Aibo has been in the field of AI robotics for over 20 years. Aibo is designed to learn and develop a unique personality based on interactions with its owners and environment, has an open architecture allowing for custom software development, and has an array of sensors to detect colors, sound, touch, and movement. It has multiple degrees of freedom to allow for realistic and complex movements. Similar to NAO, Aibo has been used in research testing HRIs as well as for medical and therapeutic uses.

#### Software and Hardware Development

The long-term goal of the SAR-VR activities is to increase social engagement, defined as a person’s activities performed within their social environment [45], to mitigate the effects of apathy in older adults. For this user-centered design study, we were interested in 3 types of interactions that could enhance social engagement. First, HHI referred to the collaborative behaviors needed between the 2 older adults to successfully complete the activity. Next, HCI, which encompasses the interactional aspects between the human user and computer [46], focused on the visual environment of the VR, the sounds, and tactile (haptic) wand feedback systems to maintain the older adults’ interest and engagement. Lastly, HRI refers to the technical aspects of the older adults and robots communicating and collaborating, as well as the psychological and social considerations of human responses and perceptions to robots [47].

**Figure 2:**
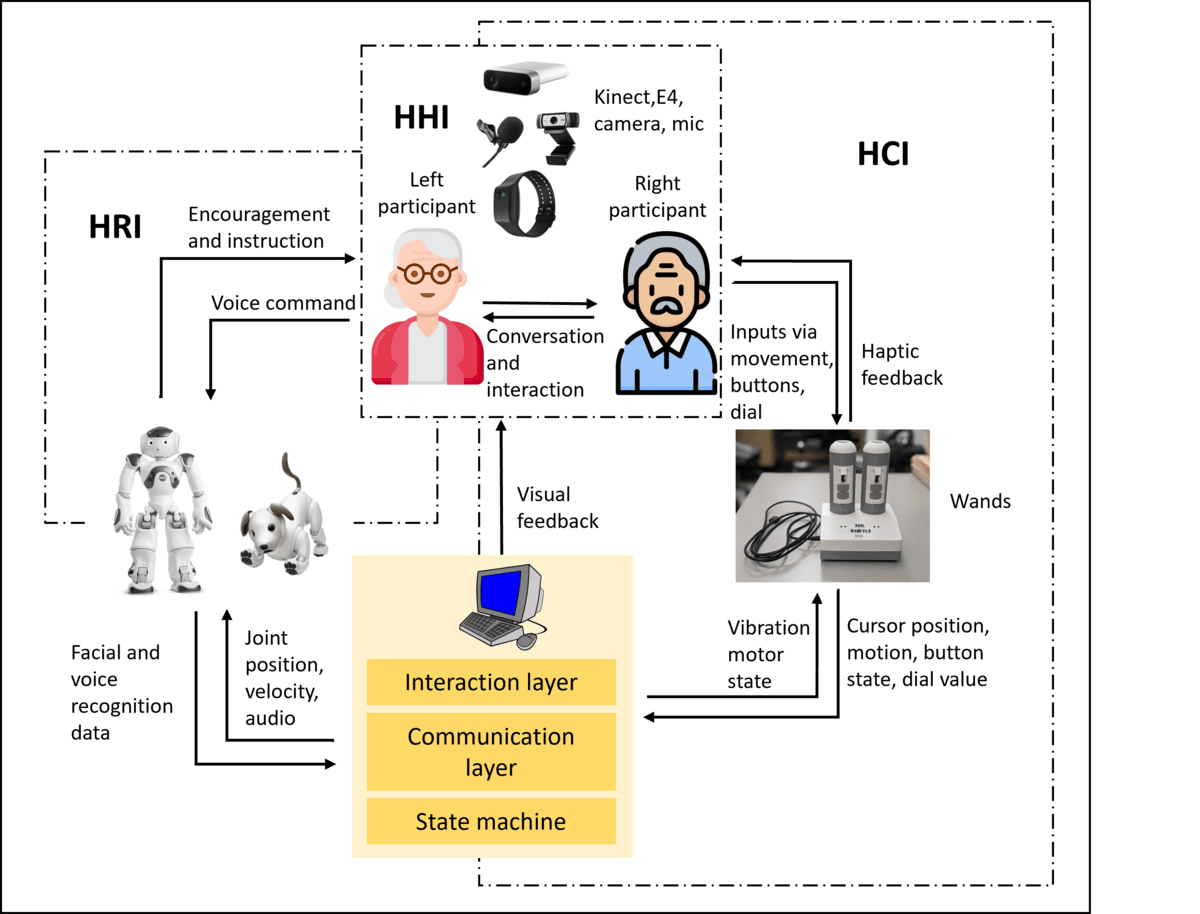
Architecture of socially assistive robot and nonimmersive virtual reality. HCI: human-computer interaction; HHI: human-to-human interaction; HRI: human-robot interaction.

Activities were designed specifically for use with a humanoid SAR and a dog SAR. The humanoid SAR acted as a coach and cheerleader, while a computer-based avatar acted as the coach and cheerleader for activities involving the dog SAR. The dog SAR then functioned as an entertainment reward. The SAR-VR engineering architecture, that is, the organized design and integration of the software and hardware system components, consisted of the 3 interactive modules—an HCI, an HRI, and an HHI module, as shown in Figure 2.

In brief, the HCI module was responsible for providing the participants with a virtual environment where they could perform the activities. The HRI module was responsible for using SARs to demonstrate the virtual activities to the participants, provide corrective feedback when necessary, encourage and motivate participants to keep them engaged, and provide rewards in the form of celebratory dance, tricks, etc, upon successful completion of the activities. The HHI module was deliberately designed to require the participants to communicate and collaborate to complete the activities since HHI is particularly effective in reducing apathy [9,11,12]. (Multimedia Appendix 1).

#### Initial Activity Choice and Rationale

**Figure 3:**
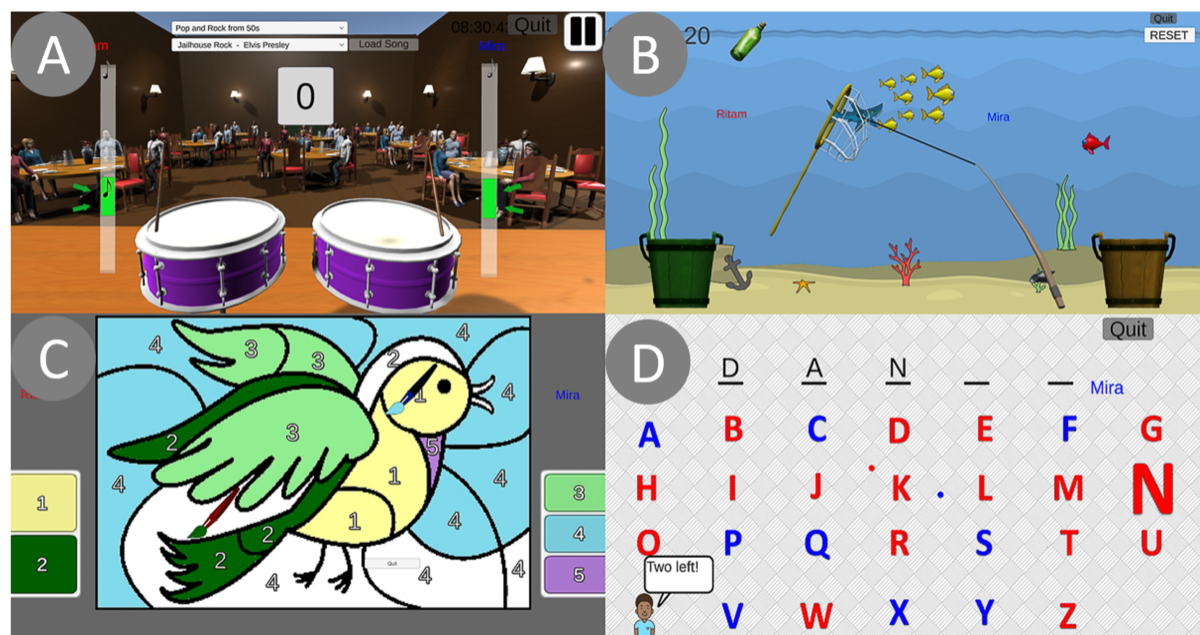
Screenshots of (A) music activity, (B) fishing activity, (C) painting activity, and (D) spelling activity.

Based on repeated brainstorming sessions and advisory panel meetings, inspiration for activities was taken from real-life activities that the older adults performed at the LTCs and hobbies they enjoyed. The interdisciplinary team determined potential virtual activities that (1) combined cognitive, physical, and social components; (2) could involve 2 older adults and encourage them to communicate and interact; and (3) were suitable for older adults with varying levels of cognitive and physical capabilities. We began development of 4 activities, 3 with Nao (music, fishing, and painting) and 1 with Aibo (spelling), as shown in Figure 3.

Music can serve as an effective tool for reminiscence and autobiographical recall [48], improve cognitive function, and reduce depression [49]. The fishing and painting activities were chosen because many older adults enjoyed these activities in their youth. Research has also shown that creative activities, such as art and music, can lead to improved mental well-being, increased focus, and a sense of calmness [50]. The virtual spelling activity was chosen since word games, such as crossword puzzles, are often enjoyed by older adults in LTCs and have shown promising results in the preservation of cognitive function [51,52].

#### Activity Design Overview

Activities were structured to incorporate physical, cognitive, and collaborative aspects (Table 1). Each activity started with an interactive tutorial to familiarize participants with the objectives of the activity, provide the opportunity to practice using the wand, and get familiarized with interacting with NAO. The wand was the custom-made human interface device, similar to a Nintendo Wii remote controller, that was designed specifically with the older adult’s physical function in mind [41,53] (for details on the wand, see Multimedia Appendix 1). NAO’s verbal and physical gestures were informed by our earlier work that enhanced older adults’ interaction with the robot. For example, at the start of each session, the engineer input the participants’ names. NAO would wave and greet each participant by name and introduce itself as “Vandy from Vanderbilt.” Ongoing positive reinforcement and feedback were directed to the individual. If one individual was having difficulty, NAO would ask the other participant to help them.

**Table 1:** Initial prototype activities, skills required, and socially assistive robot (SAR) input.

	Music	Fishing	Painting	Spelling
Description	Drumming motion with wand to play virtual drums in sync with displayed musical notes	Catch fish with virtual fishing rods using wands. Deposit captured fish into buckets	Paint-by-numbers format. Each participant has been assigned numbers with corresponding colors on palettes	A word (represents a dog command) was provided to participants. Each participant is assigned a color (red or blue) and must choose the correct letters to complete the spelling
Cognitive skills	Perception and attentional control	Problem-solving, attention, and working memory	Recognition and attention	Recall
Physical skills	Gross motor movements	Gross and fine motor movements	Fine motor skills	Fine motor skills
Social skills	Joint decision-making and participation	Cooperation and coordination	Participation	Participation and cooperation
SAR Input	NAO provides tutorials, encouragement, and reminders	NAO provides tutorials, encouragement, and reminders	NAO provides tutorials, encouragement, and reminders	NAO provides tutorials and feedback. Aibo performs the word command

Each activity had levels of increasing difficulty to accommodate varying levels of cognitive abilities among the participants (Multimedia Appendix 2). All activities underwent numerous modifications as the field testing progressed based on (1) enhancement of visual and audio characteristics and (2) meeting our goal of encouraging HHI as determined through objective and subjective feedback (Multimedia Appendix 2).

### Phase 3: Evaluation and Field Testing

#### Usability

Usability requirements of the SAR-VR activities and system were based on the intended end users—LTC older adults and staff. Data sources included observation, video recordings, and participant interviews during and after each session. Field notes, using a predetermined format, were generated after each testing session and reviewed by the research team. Videos were reviewed with the full interdisciplinary team to allow for behavioral analytics and task analysis.

We evaluated usability heuristically in 3 general areas. Contextual issues related to the environmental factors that could impact usability (lighting, sound, space issues, and furniture). Technology issues encompassed software and hardware components that resulted in errors or delays and prevented the older adults from effectively completing the tasks, potentially resulting in frustration. Activity issues were those germane to the actual activity; open-ended questions were used to obtain their thoughts on the activity, including their enjoyment, understanding, and suggestions for changes.

#### Confidence and Comfort

We examined older adults’ confidence and comfort with the system. We adapted items from the System Usability Scale (SUS) [54] since in our earlier studies, we found older adults with cognitive impairment had difficulty completing the original scale. We asked 6 structured questions to elicit their degree of confidence and degree of comfort in using the wand (2 items), interacting with the VR environment (2 items), and interacting with the robots (2 items). We modified the 5-point Likert response as a horizontal scale depicting 5 facial expressions in combination with text descriptions rather than the use of numbers, a valid self-report method for older adults who have mild to moderate cognitive impairment [55,56]. The modified scale ranged from 6 to 30, with higher numbers indicating greater comfort and confidence. We compared the first and last ratings using the paired Wilcoxon signed rank test.

#### Interaction Indices

Lastly, we examined interaction indices for HHI and HRI. Since apathy is best treated by HHI activities [11,12], we were interested in knowing the degree to which the designed SAR-VR activities encouraged HHI. At each session, we measured the rate of interactions as the number of discrete events per minute. Rates were examined over time at each session.

#### Iterative Testing and Evaluation of the SAR-VR Activities

We conducted 2 field tests in separate LTC sites using the intended deployment setup to enhance discovery of potential challenges. Activity development and testing were conducted at the first site, and validation and final refinement occurred at the second site. During each round of field testing, multiple sessions were conducted with testing and analysis that required looping back to earlier stages of the prototype development and defined requirements for cyclical refinement and improvement of both the activity and the system. The design iterations continued until users deemed that the activities and SAR-VR system met the requirements.

### Older Adult Study Procedure

Prior to the first session, baseline information was gathered from each older adult, consisting of demographic information (age, sex, education level), frequency of use of technology (smartphones, computers, pads), the Apathy Evaluation Scale-Self Rated (AES-S), and the Self-Administered Gerocognitive Exam (SAGE). The AES-S [57] consists of 18 items that measure behavioral, cognitive, and emotional domains of apathy. Each item is rated on a 4-point scale ranging from 1 (not at all) to 4 (a lot), with a total score ranging from 18 (low apathy) to 72 (extreme apathy). Although there are no standard cutoff scores to indicate clinical apathy, a score of 40-42 on the AES-S generally indicates mild apathy in older populations. The AES-S has good internal consistency (Cronbach α=.86-.94), retest reliability (*r*=0.89-0.94), and convergent and divergent validity.

The SAGE [58] assesses cognition in 5 domains—language, reasoning and computation, visuospatial abilities, executive memory, and orientation. The SAGE has good interrater reliability (intraclass correlation coefficient=0.96), specificity (88%-95%), and sensitivity (62%-95%). A score of 17-22 (maximum) suggests normal cognition, 15-16 suggests mild cognitive impairment, and <15 suggests dementia.

**Figure 4:**
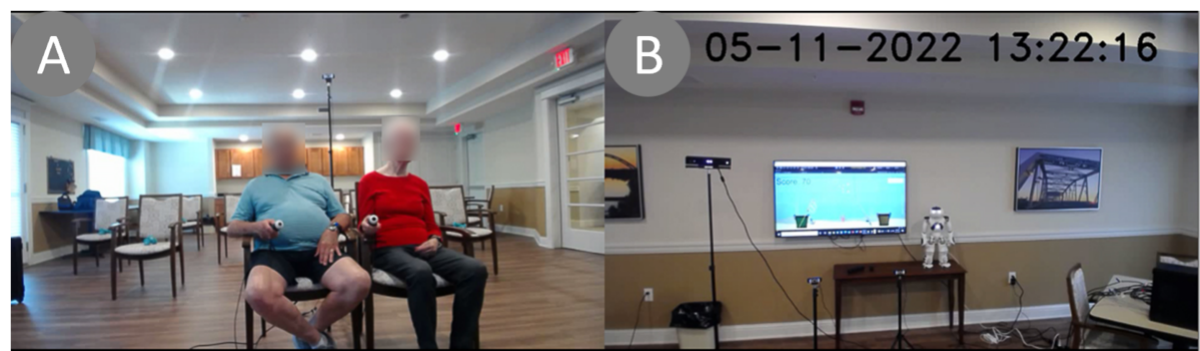
(A) Two participants performing the fishing activity (presented with permission from the participants) and (B) a virtual reality screen and NAO in front of the participants.

The same study procedure was followed at all sessions at both sites. Figure 4 shows a setup, materials, and a session in progress. Each session was attended by 3-4 researchers. Participants sat in front of and facing the system. E4 physiological sensors (Empatica Inc) were placed on both participants’ nondominant wrists. Calibration data and a 2-minute resting physiological signal were recorded for each participant. Each participant wore a clip-on microphone to record their speech. NAO was positioned in front and to the side of a large television screen. The Kinect was placed facing the 2 participants. An administrator operated the study workstation placed to the side of the participants. A total of 2 webcams were used to record the participants’ behaviors and interactions, as well as the SAR and VR environment (Multimedia Appendix 3).

Each participant participated in 5 separate sessions lasting 30-45 minutes. Each session encompassed 2 activities with a 5- to 10-minute break between activities. At the start of each activity, participants completed an orientation where they were informed of the activity and objective and how to use the wands to map their movements onto the VR environment, and provided the opportunity to practice the basic motions needed for the activity. Most sessions were completed with an older adult pair; in the event of scheduling conflicts with older adults, some sessions were completed with one older adult and a research partner. Between each session, engineers modified activities and the SAR-VR system.

### LTC Staff Study Procedure

Once the field testing was completed with the older adults, the final SAR-VR system was tested with LTC staff to determine whether they could administer the system. A total of 5 staff from Site 2 (mean age 34.2, SD 15.8 years) were recruited and consented to the study. Components of the system were set out; all components were labeled and color-coded. Staff were provided a written manual with a step-by-step guide in which components were illustrated (Multimedia Appendix 3). In addition, every section had a video guide. Staff participants were asked to set up and run the system based on the instructions. After each session, the system setup and interface were refined based on objective and subjective feedback.

### Ethical Considerations

Approval for this study was obtained from the IRBs of the research team at The Ohio State University (IRB #2020B0342) and Vanderbilt University (IRB #210386). The procedures used in this study adhere to the tenets of the Declaration of Helsinki [59] and regulations of the IRBs. The 2 types of participants were recruited for this study—older adults residing in LTC and employed staff. Safeguards were in place for both groups to ensure participation was voluntary. Older adults were informed that their decision whether to participate would have no bearing on the care received at the LTC facility, and staff were informed that their decision regarding participation had no bearing on their employment. Written informed consent was obtained from all individuals participating in the study prior to data collection and study procedures. To ensure older adults understood the study purpose, risks, and benefits, we used the University of San Diego Brief Protection of Human Subjects Capacity to Consent instrument [60]. If a participant could not demonstrate understanding, they were ineligible. Research assistants underwent training to recognize signs of frustration, anxiety, or stress displayed by participants; any sign of discomfort resulted in termination of the session. Research assistants were trained in procedures to protect privacy and confidentiality. Participants were assigned unique study identification numbers, and all deidentified data were stored in a secure database and password-protected. Participants received financial compensation for study activities, including the interactive sessions and data collection procedures, to acknowledge their time and reduce attrition. An external safety officer, designated by the National Institute on Aging (NIA), reviewed and approved the study protocol and informed consent documents prior to the start of the study. Biannual reports were provided to both the safety officer and the NIA program officer to review progress and any untoward events.

## Results

### Older Adult Profile

A total of 12 older adults participated (Table 2). In the first field study, 6 older adults initially consented to participate. A 3-week delay occurred between consent and implementation due to a COVID-19 outbreak in the facility. Subsequently, 2 participants chose not to continue with the study, having lost interest over time; both these participants had significant cognitive impairment. The 4 remaining Site 1 participants (mean age 85, SD 9.3 years; mean AES-S score 29.3, SD 5; mean SAGE score 11.5, SD 3.6) completed all sessions. In Field Testing 2, eight residents (mean age 80, SD 4.7 years; mean AES-S score 26.35, SD 2.7; mean SAGE score 14.3, SD 4) were eligible, consented, and participated. Of the total sample, 4 participants had no experience with technology. Self-reported apathy scores were below the 40-cutoff point. Cognitive screening revealed that 3 (75%) of the 4 older adults at Site 1 had scores indicating dementia, whereas only 3 (38%) of 8 older adults at Site 2 had scores indicating dementia.

**Table 2:** Older adult characteristics for Field Testing 1 and 2.

Participant ID		Age (years)	Sex	Education	Technology use	AES-S^a^ score	SAGE^b^ score	Completed the study
**Field Testing 1**								
	A1001	89	Female	High school	Everyday	25	11	Yes
	A1002	90	Female	High school	Never use	32	17	Yes
	A1003	92	Male	Graduate degree	Everyday	26	8	No
	A1004	92	Male	Bachelor’s degree	Everyday	24	11	Yes
	A1005	69	Male	Graduate degree	Never use	36	7	Yes
	A1006	89	Female	High school	Never use	25	5	No
**Field Testing 2**								
	A1007	82	Male	Bachelor’s degree	Everyday	26	18	Yes
	A1008	81	Female	High school	Rarely	25	18	Yes
	A1009	75	Female	Graduate degree	Everyday	28	18	Yes
	A1010	80	Male	Bachelor’s degree	2-3 times a week	31	11	Yes
	A1011	80	Female	Graduate degree	Never use	29	6	Yes
	A1012	72	Female	Trade school	Everyday	25	12	Yes
	A1013	81	Female	Bachelor’s degree	Everyday	22	16	Yes
	A1014	89	Female	Graduate degree	Everyday	24	15	Yes

^a^AES-S: Apathy Evaluation Scale-Self Rated. Higher scores indicate greater apathy.

^b^SAGE: Self-Administered Gerocognitive Exam. A score of 17-22 (maximum) suggests normal cognition, 15-16 suggests mild cognitive impairment, and <15 suggests dementia.

### Usability: Contextual, Technological, and Activity Issues

Attention to the environment was necessary. At the first site, older adults had difficulty seeing the monitor because of glare; subsequently, blinds needed to be drawn at all sessions. The voice of NAO was difficult for older adults to hear and understand clearly. After several trials of altering its pitch, style, volume, and speed, we found a setting that was the most clearly understood (Multimedia Appendix 1). Early on, we observed that older adults became fatigued with holding and manipulating the wands. They suggested having chairs with armrests and sitting at a table would provide them with the opportunity to rest their arms. The VR environment underwent a number of changes to enhance the visualization, including adjustments to the size, color, and placement of components.

Technology issues occurred in both the hardware and software components. Originally, NAO was programmed to do a short dance to celebrate the successful completion of an activity. After one of the NAO fell off the table and broke its hip, we elected to forgo that component despite the older adults’ obvious enjoyment. NAO would give feedback based on the queuing of each observation of the older adults’ performance, either positive or corrective. This resulted in a long list of feedback statements, ultimately causing a delayed response that did not correspond to the older adult’s current performance. The mismatch of feedback with current behavior caused confusion and, at times, frustration. NAO was reprogrammed to provide feedback only after an older adult performed an errant behavior 3 times in a row. The customized wands were remodeled several times to enhance grip and controls; wands were also color-coded to match the person’s cursor color in the VR environment.

Throughout the study, older adults provided suggestions to enhance the activities to make them more enjoyable. Table 3 highlights major changes that occurred during Field Testing 1 and 2.

**Table 3:** Changes to activities to enhance older adults’ engagement.

Component			Challenges		Changes
**Field Testing 1**					
	Music activity	Sometimes, only one participant got consecutive notes while the other had to wait for extended periods. Sometimes NAO’s consecutive feedback was too frequent and overwhelming.		Note generation changed to “alternate” instead of “random.” Increased the length of the time between NAO’s feedback statements.	
	Fishing activity	Minimal collaboration was required between the 2 participants, who both had fishing rods.		The initial activity of 2 fishing rods was replaced by 1 fishing rod and 1 net. The participant with the rod was responsible for catching a fish and passing it to the participant with the net. The participant with the net had to help guide the net to the fishing rod for the fish to be released, and then deposited the fish into the bucket. This increased collaboration and communication.	
	Painting activity	Participants had difficulty seeing the color of the cursor “dot” and knowing which color was selected.		The cursor changed from a “dot” to a “paintbrush,” with the tip changing color to reflect the color that was selected.	
	Spelling activity	Some of the participants did not realize it was their turn to pick the letters and would keep waiting. Sometimes, the participants forgot what the target word was. Some participants had difficulty locating the correct letters among the rows of letters.		A reminder was added for NAO to cue participants when it was their turn to pick a letter. A hint was added to the top of the screen to remind both participants of the word they are supposed to spell. A slider was added that controls the number of excess letters visible on the screen. This allowed the administrator of the system to reduce visual stimuli overload and make it easier to locate the correct letter.	
**Field Testing 2**					
	Music activity	Some participants were able to predict when it would be their turn to hit the drum and complained that it was boring.		Alternate assigned notes were replaced with a probability approach to minimize predictability, yet ensuring each participant had sufficient notes to play.	
	Fishing activity	Both buckets on the screen could accept fish, which minimized the amount of attention and movement by the participant with the net.		Only 1 bucket at a time was active in the highest difficulty level. This required the participants to attend to which bucket was “active” during their play and provided the participants with the net more opportunities to play.	
	Painting activity	Some participants had difficulty choosing a color from the palette, designed with 2 columns of colors.		The palette layout changed to 1 column.	
	Avatar for dog activities	Participants’ attention was divided between the 2 robots.		A screen avatar replaced NAO to provide instructions and feedback.	

### Comfort and Confidence Ratings

**Figure 5:**
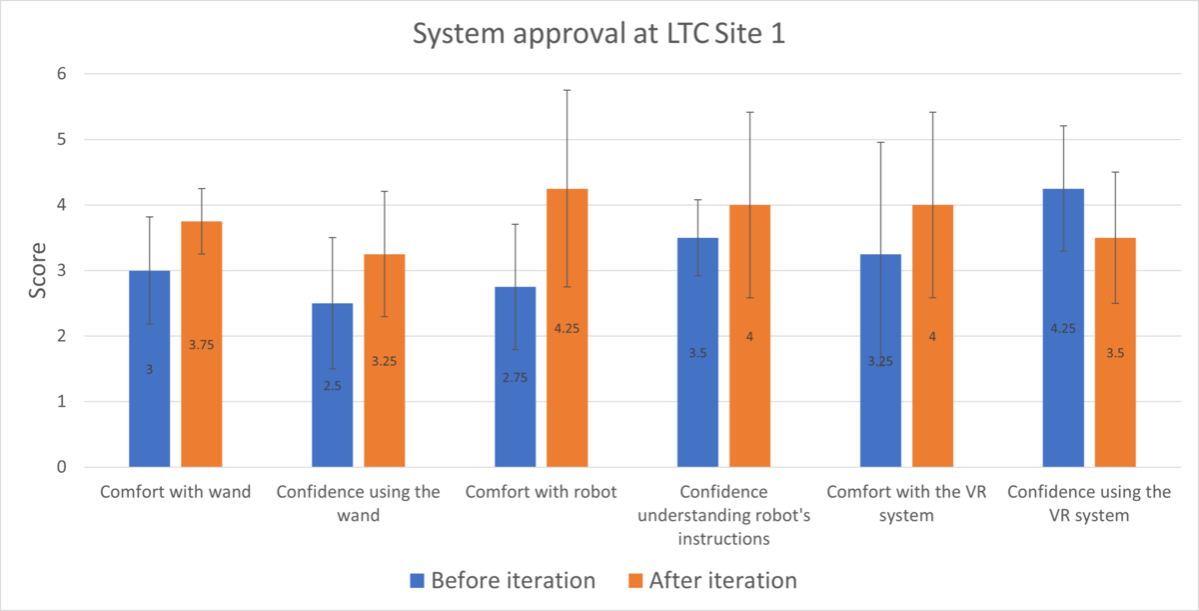
Site 1 older adults’ rating of confidence and comfort with the socially assistive robot and nonimmersive virtual reality (VR) system elements from the first to last session. LTC: long-term care.

After each session, participants at both sites rated their degree of comfort and confidence in (1) using the wands, (2) interacting with the robot, and (3) interacting with the VR environment. Between the first and final sessions, participants at Site 1 increased their comfort and confidence in 5 of the 6 categories. The average increase across all 6 categories was 0.58 (95% CI 0.04-1.12; Cohen *d*=0.45; Wilcoxon signed rank test *P*=.03). The category-wise mean improvements and standard deviation for each item are displayed graphically in Figure 5 (see Multimedia Appendix 4 for numerical values).

**Figure 6:**
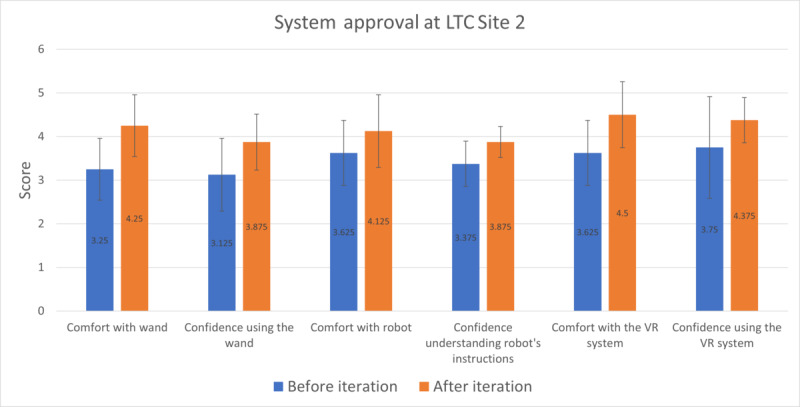
Site 1 older adults’ rating of confidence and comfort with the socially assistive robot and nonimmersive virtual reality (VR) system elements from the first to last session. LTC: long-term care.

Participants at Site 2 showed enhanced comfort and confidence with an average increase of 0.71 (95% CI 0.43-0.99; Cohen *d*=0.73; Wilcoxon signed rank test *P*<.001) between the first and final sessions. The category-wise mean improvements and standard deviations are shown in Figure 6 (see Multimedia Appendix 4 for numerical values).

### Interaction Indices: Participant-Participant, Participant-Robot, and Participant-Researcher

Analysis of the recorded videos revealed that the average interaction of older adults with their partner increased steadily across sessions, with iterations from 0.16 interactions per minute during the first set of sessions to 1.14 interactions per minute in the final set of sessions. The average number of robot interventions decreased from 1.10 per minute to 0.73 per minute. The average number of researcher-assisted interventions decreased from 0.34 per minute to 0.10 per minute. These results suggest an increased engagement and interaction among older adults and an increasing effectiveness of the system with each iteration.

### Responses to Open-Ended Questions

Participants were asked open-ended questions (Multimedia Appendix 4) about their experience with the activities. Participants reported they enjoyed the activities and expressed interest in playing them again. They described the games as fun and interesting, noting that once they became familiar with the mechanics (eg, casting the fishing rod), the experience was enjoyable and engaging. They also indicated that the robot’s instructions were clear and sufficient, requiring no additional guidance.

### Staff Setup of the SAR-VR System

A total of 5 staff members evaluated the system from the perspective of ease of use and convenience. They set up and ran the system following written instructions prepared by the researchers. The first 2 participants who worked with the system had difficulty following the steps in the correct order and required prompting from the research staff. To remedy this, the instruction manual was divided into 3 parts, that is, presession, during-session, and postsession. The presession consisted of initialization and calibration of components; during-session procedures involved running the system, activity selection, and changing the activity difficulty levels; and postsession procedures consisted of securely closing all applications and sensors, saving the data, and turning off the system. This division helped avoid confusion and led to a smoother setup by other participants. All the staff participants commented that the first-time setup had a learning curve, but they would feel more confident if they were to set up the system again. Overall, they found the integration of the different components of the system to be satisfactory and user-friendly.

### Challenges in the UCD Process

#### Accessing Older Adult Participants

A major challenge to the UCD process was the ongoing COVID-19 pandemic. The study began in May 2020, at the start of COVID-19. Universities and LTC sites were on lockdown for months. The health care system was severely strained, delaying our access to experts from LTC sites. We were unable to conduct initial prototype testing in the Vanderbilt University Engineering Lab as initially planned, since older adults were kept in social isolation. The ongoing UCD was interrupted several times as COVID-19 waves continued to occur from 2020 to 2022, restricting access to LTC sites and older adults.

#### Working With Older Adults in LTC Sites

Most older adults had cognitive deficits that at times impeded their ability to interact with the system; by offering various levels of difficulty, we found that all participants could complete each activity. For some older adults, physical impairments, such as arthritis, made it difficult to handle the input device, and fatigue occurred easily. Subsequent changes to the wand facilitated grip and controls. Keeping activities to less than 20 minutes and the use of furniture supports eased the onset of fatigue. Visual impairments impeded their ability to see the VR environment. Ongoing adjustments to the color, size, and spacing of the elements resolved this issue. Hearing impairments impeded their ability to hear NAO’s voice, and multiple changes were made until we were able to find a satisfactory pitch and volume. (see Multimedia Appendices 1 and 2 for refinements based on aging changes).

#### Working Within the LTC and With Staff

Logistics of the UCD proved challenging at times. Because we needed to take the equipment to the LTC, versus having the older adults come to the engineering laboratory, we needed to coordinate our schedules. We found that scheduling the sessions was complicated by competing priorities within the LTC, staff illnesses and shortages, and competing use of the activity room. The physical setup took additional time due to internet issues (that had not been an issue at the engineering laboratory).

### Physiology and Motion Data for Future Research

Multimodal physiological and behavioral data were collected during the sessions to inform future research and system development. Physiological signals, including blood volume pulse, electrodermal activity, skin temperature, and accelerometer data, were recorded using the E4 sensor. These data can be analyzed to infer participants’ affective states (eg, stressed vs relaxed, engaged vs disengaged) by examining the fluctuations in their autonomic responses during interaction with the system. Such insights can guide the design of adaptive features that adjust task difficulty, robot behavior, or feedback strategies in real time to better match the user’s emotional and cognitive state.

Joint position and head orientation data were captured using the Kinect sensor. These data enable the quantification of motor performance (eg, range of motion, smoothness of movement) and the assessment of social engagement (eg, frequency of head turns toward the partner or robot, use of gestures, changes in posture). This allows for objective evaluation of both physical rehabilitation potential and the quality of social interaction.

Finally, time-stamped performance logs provide a record of task success, errors, and completion times, which can be correlated with physiological and kinematic measures. Together, these multimodal datasets will allow for richer models of user engagement, support longitudinal monitoring of progress, and inform further refinement of SAR-VR interventions for older adults.

## Discussion

### Overview

In this user-centered design study, we used the literature, our clinical expertise and prior research, and an advisory panel that included older adults to identify activities that could be created for older adults residing in LTC settings using a combined SAR and nonimmersive VR system (SAR-VR). The SAR-VR system was designed to engage pairs of older adults with the long-term goal of using this technology in LTC settings for those with apathy. The underlying premise is that those with apathy are best treated by activities that are performed with others and that combine cognitive and physical aspects [11,12]. We conducted numerous sessions with LTC older adults, who had varying levels of cognitive and physical impairments, to achieve the development and validation of 4 SAR-VR activities for this specific population. We are currently testing the effectiveness of the SAR-VR system to mitigate apathy in a multisite randomized controlled trial.

Although others [61,62] have highlighted the importance of UCD with older adults and adapting processes for this population, few studies have actually involved older adults with cognitive impairment in the design of technology [34,39]. Second, it is important to involve staff in UCD such that they feel comfortable operating new systems and thus ensure system scalability in LTC settings. Thus, we focused on the 2 key stakeholders, LTC older adults and LTC staff, in our UCD of the SAR-VR system and activities. Our findings demonstrated the effectiveness of an iterative UCD approach for designing SAR-VR activities and systems for older adults in LTC settings.

The study provided valuable lessons about conducting UCD of SAR-VR systems in LTC settings. First, the timeline for development and testing was prolonged due to multiple waves of COVID-19, virtually shutting down access to the older adults. Second, as is often the case in the design of technology, our initial design decisions were based on our expertise and experience, the literature, and experts in the LTC field. Some of these decisions were discovered to be incorrect during field testing. For example, we designed the graphics to be seen easily by the older adults (contrasting colors, font size) and lowered the pitch of NAO’s voice to accommodate age-related vision and hearing changes [63]. However, we found our graphics required further modifications, and a series of voices had to be tested before older adults agreed to the pitch, tone, and speed. The initial creation of the handheld wand controller also required several changes to accommodate older adults’ grips, maneuverability, and vision. Third, accessing older adults in the LTC sites proved more challenging than anticipated. It was not uncommon for staff to cancel and reschedule our sessions based on their availability, competing demands, or illness among the older adults—all of which further prolonged the process.

Despite these challenges, we found the UCD process successful in developing the SAR-VR system to deliver multimodal activities for older adults with cognitive and physical impairments. With the increasing population of older adults with dementia and insufficient skilled care staff, technological interventions are a promising strategy in the delivery of care processes. We succeeded in developing an innovative SAR-VR system that could deliver a variety of activities that combined physical, cognitive, and social domains for pairs of older adults residing in LTCs. Such multidomain stimuli, along with HHI, have been shown to be effective in reducing apathy [9,11]. Each activity required the participants to cooperate and make joint decisions, thus encouraging interaction and conversations. While other work has attempted to reduce apathy using immersive VR technology [64,65], research on developing technology to foster and mediate natural HHI among older adults with minimal caregiver intervention is scarce. Out of the total 14 participants recruited for the study, 12 stayed for the full duration of the study. Participants’ confidence and comfort with the SAR-VR improved over time for both those who conducted the early development work (Field Testing 1) and the validation work (Field Testing 2); overall, the participants enjoyed doing the activities.

Since the system is designed to be deployed at LTCs, we tested the setup procedure of the system with LTC staff to ensure ease of use. All enrolled staff could set up and run the system with the instructions provided without any special training, thus proving the practicality and deployable nature of the system in an LTC. When asked if they would be more comfortable if they were to set up the system again, most replied in the affirmative and mentioned that the most challenging part was identifying components that they were otherwise unfamiliar with. The iterative design process allowed us to make modifications to the various components of the system, including the setup guide, until the system was suitable and enjoyable to both target populations, the older adults and the LTC staff.

While UCD has been used to develop immersive VR [39], eHealth applications [66,67], exercise games [68-70], and websites [71,72] for older adults, to the best of our knowledge, this is the first study to take an iterative UCD approach to developing a SAR-VR system and activities for pairs of older adults. The resultant modular architecture and activities set the foundation for investigating their effectiveness in mitigating apathy among older adults with dementia. We designed the system to easily be adapted to other robots, activities, and interaction devices. The intensity of the physical, cognitive, and social components of the activities can be individually tuned, as desired, to study the effect of each component.

Physiological, motion, and performance data collected from the study will provide insights for further analysis and refinement. Physiological data can provide insights into the level of stress and cognitive load of the participants during performance of the activities, which can later be used to design an affective state–based closed-loop system to automatically regulate the difficulty of the activities. The joint position data from the Kinect sensor can later help automate the detection of nonverbal communication by tracking head pose and gestures.

### Limitations

This work, although promising, is not without limitations. First, it is limited by its small sample size and purposive sampling, as well as the short duration of the study procedures, that is, 5 sessions. Small sample sizes are typical in initial UCD studies given the need to facilitate rapid iterative testing, as well as keep personnel and financial resources within reasonable limits [38]. A larger study of longer duration is required to gauge user perception accurately and mitigate the novelty factor. Our target population is older adults who reside in LTC, with apathy, and with cognitive or physical impairments; our sample did not have evidence of clinically significant apathy, and several were screened with normal cognition. The user perception of the system depends on several factors, such as cognitive abilities, previous exposure, and familiarity with similar technology, as well as personal preferences toward particular activities. A larger sample size is required to generalize the preferences, as data from smaller samples are easily skewed by outlier individuals. A larger sample size would also allow for the identification of personal characteristics associated with successful and ongoing involvement with the SAR-VR system. Second, we used commercially available robots, thus limited by the physical attributes of the robots. For example, we found that we needed multiple adjustments to the humanoid robot’s voice that would facilitate older adults’ ability to hear and understand its language. We had to position the humanoid robot onto a table to facilitate the visual field of the older adult who was focusing on the virtual environment displayed on a television or monitor. Nevertheless, we chose robots that have been available for many years to researchers because of their ease and versatility for customized programming. Lastly, we created only 4 activities during this study. We have since designed several more activities based on our experiences with this study that will allow for more variety in future studies.

### Conclusions

Building upon our previous work on SARs and VR, this work details the use of user-centered iterative design techniques and processes to develop an innovative SAR-VR system that can provide activities that consist of physical, cognitive, and social aspects and are specifically designed to promote HHI through activities that require cooperation between participants. We created 4 activities—3 humanoid robot–based activities, where the humanoid robot NAO acted as the coach to provide hints and corrective feedback and mediate the interaction between older adults, and 1 animal robot–based activity, where an avatar in the VR environment provided the feedback and the puppy robot Aibo performed tricks as rewards to incentivize the older adults to complete the activity. A finite state machine was developed to provide appropriate real-time feedback in response to participants’ performance in the activities. The system also collected video, physiology, joint position, and performance data that will be used for future investigations into how stress and engagement patterns in older adults varied with activity difficulty level. We incorporated various tunable difficulty levels in the activities to accommodate different physical and cognitive abilities. A variety of activities using both the humanoid and animal robots were developed to enhance engagement and reduce monotony. The system has a simple user interface and an easy-to-follow setup guide for the LTC staff. Currently, we are conducting a multisite randomized controlled trial [73] to compare the effect of usual care to the SAR-VR plus usual care on reducing apathy (primary outcome) among 188 older adults with cognitive impairment and enhancing executive cognitive function (secondary outcome). Eligibility of state-licensed LTCs includes an hour’s drive from the principal investigators’ offices, certification by the US Centers for Medicare and Medicaid, and physical space to store the research equipment. Eligible older adults are those aged 65 years or older, with 3 months or more of residence at the LTC, evidence of mild to moderate cognitive impairment, and symptoms of apathy. The intervention takes place twice weekly for 8 weeks; 4 weeks participating in NAO activities and 4 weeks participating in Aibo activities. Data are collected at baseline, each session, and post experiment. Findings will aid in determining the effectiveness of the SAR-VR system and identifying characteristics of the system, activities, and older adults that are associated with effectiveness. We are also gathering information to identify barriers and facilitators to implementation; this knowledge is needed to address the scalability of the intervention, including the training needs of staff.

## Appendix

Multimedia Appendix 1 Details of the socially assistive robot with nonimmersive virtual reality system architecture.

Multimedia Appendix 2 Description of socially assistive robot with nonimmersive virtual reality activities.

Multimedia Appendix 3 Study setup guide.

Multimedia Appendix 4 Questionnaires and results.

## Abbreviations

AES-S: Apathy Evaluation Scale-Self Rated
HCI: human-computer interaction
HHI: human-to-human interaction
HRI: human-robot interaction
IRB: institutional review board
LTC: long-term care
NIA: National Institute on Aging
SAGE: Self-Administered Gerocognitive Examination
SAR: socially assistive robot
SAR-VR: socially assistive robot and nonimmersive virtual reality
SUS: System Usability Scale
UCD: user-centered design
VR: virtual reality
