# Supplementary material for: Engaging Older Adults and Staff in the Co-Design and Evaluation of Socially Assistive Robot and Virtual Reality Activities for Long-Term Care: User-Centered Study
Source: JMIR Aging. 2025 Dec 2;8:e75288. doi: 10.2196/75288 (PMC12709162; doi:10.2196/75288)
Supplement: Multimedia Appendix 1 [file aging_v8i1e75288_app1.docx]

Multimedia Appendix 1

**Engaging Older Adults and Staff in the Co-design and Evaluation of Socially Assistive Robot and Virtual Reality Activities for Long-Term Care: A User-Centered Study**

**Details of the SAR-VR System Architecture**

# Overview:

### SAR-VR System Architecture

The long-term goal of the SAR-VR system activities is to increase social engagement (i.e., Human-Human Interaction (HHI)) among older LTC adults with apathy through collaborative activities mediated by Human-Robot Interaction (HRI) and Human-Computer Interaction (HCI). To achieve this, we designed a system architecture (i.e., framework) that encourages two participants to perform an activity together in a non-immersive virtual environment. Activities were designed specifically for use with a humanoid SAR and a dog SAR. The humanoid SAR acted as coach and cheerleader, while a computer-based avatar acted as the coach and cheerleader for activities involving the dog SAR. The architecture consists of three major modules: an HCI, an HRI, and an HHI module, as shown in Figure 2.

**Figure. SAR-VR Architecture**


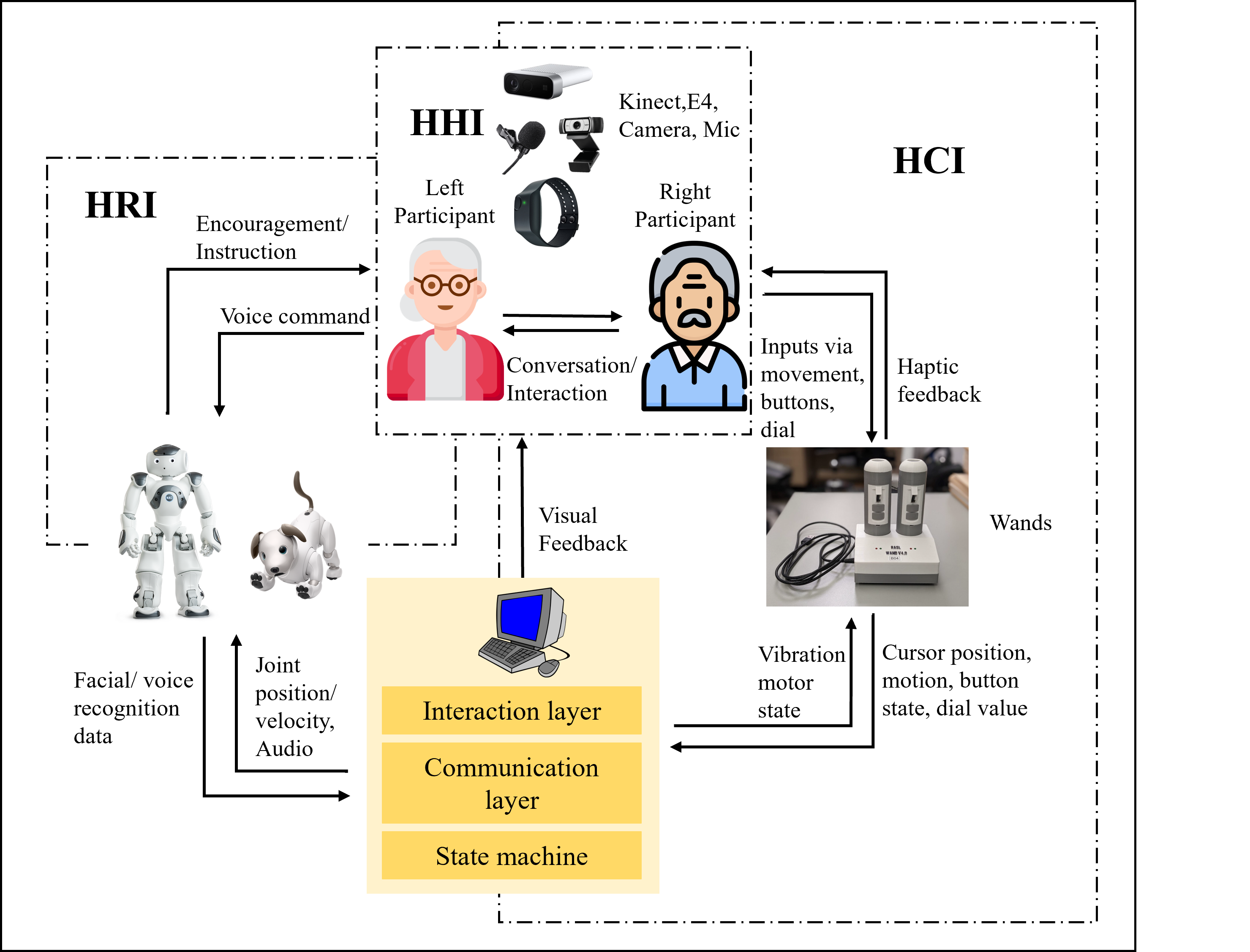


The *HCI module* is responsible for providing the participants with a virtual environment where they can perform the activities. It consists of a Windows computer running the VR environment designed using the Unity game engine and a set of two ‘wands’, one for each participant, which are custom-designed controllers to interact with the virtual activities. The wands function similar to Wii remote controllers; they contain inertial measurement units (IMU), which tracks the participant’s hand motion and control virtual objects on the screen, a vibration motor to provide tactile feedback and two buttons to select objects. They communicate with the computer wirelessly via a dongle using the ESP-NOW protocol. A finite state machine controls the different states of the activities.

The *HRI module* is responsible for using SARs to demonstrate the virtual activities to the participants, provide corrective feedback when necessary, encourage and motivate participants to keep them engaged, and provide rewards in the form of celebratory dance, tricks etc. upon successful completion of the activities. The HRI module can use two SARs, a humanoid robot Nao and a puppy robot Aibo. The HRI module communicates with Nao via a socket connection wirelessly through a router and with Aibo through an Application Programming Interface (API) key. In our earlier work, we found that older adults varied in their responsiveness and enjoyment of the two types of SARs; using two different forms will allow us to examine the characteristics of older adults best served by SAR type for future applications. The HRI module enables communication between the HCI module and the SARs. Nao acts as a coach and guides the participants through interactive tutorials before each VR activity. Nao also provides encouragement and corrective feedback to keep the participants engaged throughout the VR activity. Aibo entertains the participants and incentivizes the completion of VR activities by performing tricks as rewards for activity completion. In the final iteration, for the activity with the dog robot, an on-screen avatar acts as a coach and delivers encouragement and feedback through the VR environment.

The *HHI module* was deliberately designed to require the participants to communicate and collaborate to complete the activities since HHI is particularly effective in reducing apathy. HHI, in the form of non-verbal communication, is measured using participants’ body position and orientation data collected using a Microsoft Kinect sensor; verbal communication is measured from video recordings of the sessions. Multimodal physiological data are collected using the Empatica E4 sensor to inform future studies that may provide an insight into the participants’ physiological state during the performance of the activities. The audio, video, and data from all the sensors are synchronized using time stamps. A user-friendly menu system enables easy navigation of the system and selection of activities.

# Wand design

The wand is a custom designed HCI device that is used to manipulate the virtual environment. From our literature survey on HCI devices for older adults, we found that a controller using both button and motion-based control was the most suitable. Most such controllers available on the market are designed for gaming and not suitable for older adults in terms of weight, grasp, and ease of use. We consulted with an occupational therapist specialized in geriatrics during the design of the wand. The wands feature an ergonomic grip design that can accommodate a variety of palm sizes. The wand dimensions were determined based on guidelines from the Canadian Center for Occupational Health and Safety. The wand is able to control the position of a cursor on the screen, has buttons and a dial for input, that are suitable for older adults. It uses an inertial measurement unit (IMU) to determine its orientation. It also has a vibration motor for haptic feedback. Four versions of the wands were developed over the course of this study; the latest version is shown in Fig. 1. It features an ESP32 microcontroller core from Espressif, ICM20948 IMU from InvenSense, wireless communication, and contact charging.


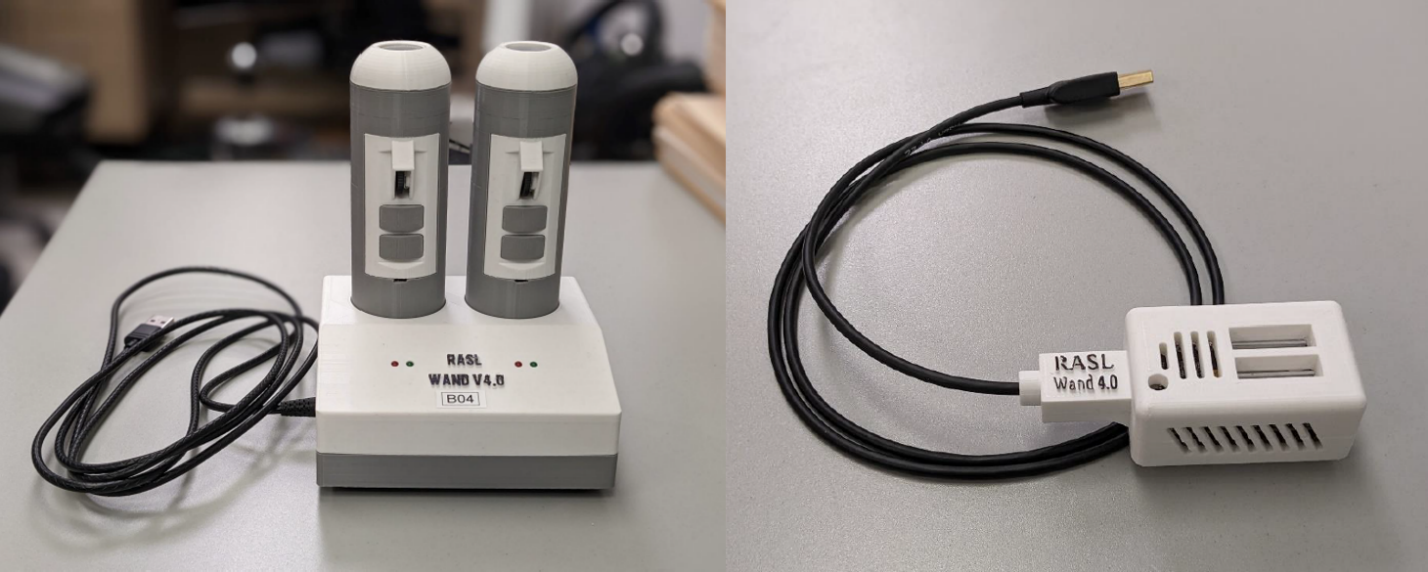


Figure: Left – Wands with charger, right – wireless receiver

# Finite State Machine

Different methods are available for modelling control systems. A Finite State Machine (FSM) was selected as it provides a simple and concise way to structure a control system where the behavior of the system is governed by a set of predefined conditions. An FSM is best suited for modelling systems that exhibit discrete sequential behavior. It has a finite number of states, conditions to transition between the states, and actions associated with each transition. In an FSM model, the system can only be at one state at a time.

When the system first turns on, the state machine is in the “Start State” where preliminary checks are conducted to ensure all components are connected to the system. Once verified, the system enters the “Stable State”. This state conducts the regular flow of the activity. Each activity has a set of defined states and corresponding state transition conditions. Fig. 2 shows the block diagram of a state machine for the Music Activity.


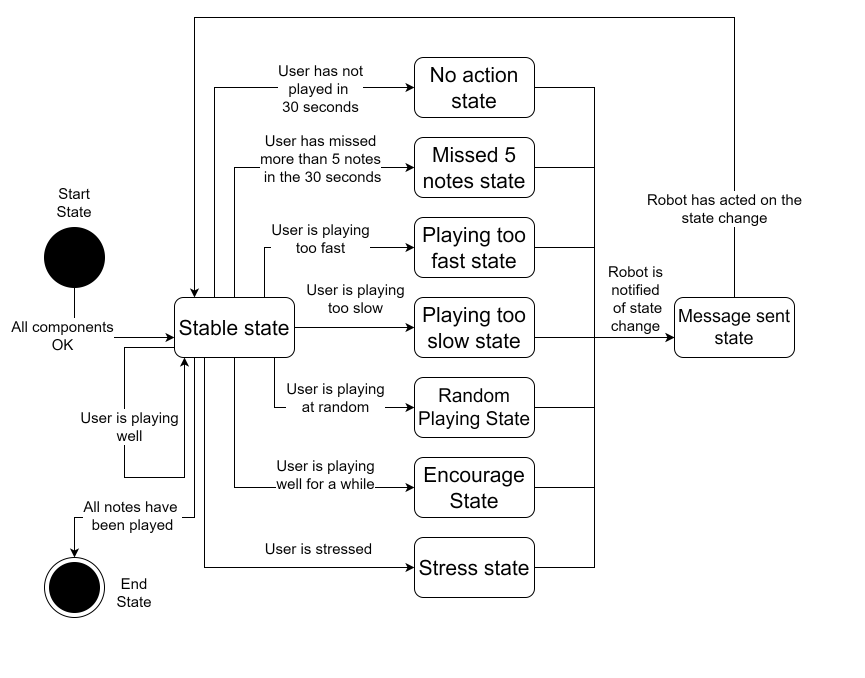


Figure: Block diagram of state machine for Music Activity

To understand how the FSM works, let’s walk through an example. After verifying all components, the system enters a Stable state and continuously scores timing and accuracy against the active rhythm. If the participant is inactive for 30 s it transitions to No-action; if more than five notes are missed within a 30 s sliding window it moves to Missed-5-notes; tempo deviations route to Playing-too-fast or Playing-too-slow; highly erratic hits trigger Random-playing; sustained good performance enters Encourage; and a stress flag enters Stress. Any such transition notifies the coach/robot and moves through Message-sent, where a brief cue or praise is delivered. A short cooldown in Message-sent prevents back-to-back prompts; afterward the machine returns to Stable to re-evaluate with refreshed 30 s windows. If performance recovers (e.g., several on-time hits), the FSM clears the alert state automatically; if it persists, it can revisit Message-sent to escalate guidance. When all notes in the sequence have been played, the machine exits to the end state and logs the session summary.

# Robot communication layer

The system was developed to be flexible with various makes of robots. Feedback can be conveyed from any virtual avatar or robot and will only require changes to one intermediate layer. This intermediate layer is used to translate human-understandable, English messages such as "LeftPlayingFast" to robot-specific messages. This design allows for easy exchange of the type of robot used with minimal change in software. Only the robot side and the communication layer will need to be modified and the activity itself will not need any modification. For example, if Nao needs to be replaced by some other humanoid robot, the state machine output will remain the same. The communication protocol supported by the new robot will be added to the communication layer and the functional behaviors will have to be installed in the new robot. Fig. 3 shows the block diagram of the communication layer.


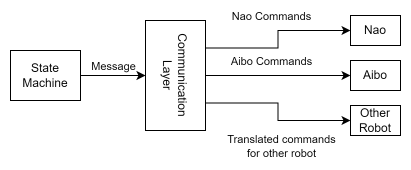


Fig. 3: Block diagram of communication layer

# Feedback animations and speech

The humanoid robot Nao is used to provide instructions and feedback to the participants during the activities. During the initial design of activities and meetings with older adults, multiple voices were generated for Nao using play.ht (https://play.ht), an online AI speech synthesizer. The pitch, speed, and pace of speech were adjusted until the older adults provided a consensus on the preferred speech domains. The final parameters chosen are mentioned below.

Voice: Guy, Male, English

Voice Style: mix of Regular, Cheerful, Excited, Friendly

Frequency: 48 KHz

Speed: 80%

Nao’s animations and speech fall into three major categories: instruction, corrective feedback, or celebrations. Instructions are typically given during the tutorial and throughout the activity if a new component is introduced. The corrective feedback is prompted when a participant is either not completing part of the activity or is struggling to complete the required action. Corrective feedback is designed to always be positive and encouraging while providing reminders or hints to the participants. Each activity has activity-specific feedback and celebrations. There are positive encouragements that are interspersed throughout all activities. Each speech component has corresponding movements programed for Nao using Choregraphe (www.softbankrobotics.com). The movements are designed to match what is said and to mimic typical human body language. If Nao is directing the participants’ attention to the screen, it will use its arms to gesture towards the location of the element it is referencing.

Aibo has several built-in tricks and functions from Sony (us.aibo.com), such as sit, dance, and rollover. Using the web API, the state machine can call specific actions to have Aibo perform tricks during the dog related activities.

# Data collection

## Video recording

Video data were collected to examine participant behavior and response to robot interactions (instruction, feedback, and celebration). Two cameras were used, one to record the participants and the other to record the robot. A custom video recording tool was created using Python3 and OpenCV to simultaneously record two monitors and two cameras with synchronized audio and time stamps. This composite video can be used to label participant behavior for future machine learning applications and for diagnosing system issues. Recordings were coded using the Noldus Observer XT® system (Noldus Information Technology, Netherlands) for analyzing behaviors utilizing a coding scheme to identify dyadic, human-computer, and human-researcher interactions.

## Performance data

The system generated time-stamped text logs recording the performance of the participants on the activities, the response of the system and robot feedback. These logs can be analyzed to gain further insight into the performance of the participants and check the effectiveness of the system.

## Joint position and posture data

The Kinect v2 sensor from Microsoft is used to capture the joint position of each user through a Python program. For each user, we are able to capture 32 different joint orientations and positions with a sampling rate of 30: Pelvis, Spine navel, spine chest, neck, clavicle left, shoulder left, elbow left, wrist left, hand left, hand tip left, thumb left, clavicle right, shoulder right, elbow right, wrist right, hand right, hand tip right, thumb right, hip left, knee left, ankle left, foot left, hip right, knee right, ankle right, foot right, head, nose, eye left, ear left, eye right, and ear right. The orientation and position information of each joint is saved to a JSON file where the keys are the timestamp at which the sample was collected.

At each sample, the program scans the number of people in the frame and then collects joint information about each. By default, the Kinect sensor can capture information up to 4.5 meters away; we limited the range to 2.5 meters as the users will be sitting close to the sensor. This allowed us to ignore people walking in the background. Additionally, if there are more than two people detected within the range of interest, the sensor stops collecting information. With only two people in the frame, the program can differentiate between the two users through their relative positions.

## Physiology data

The E4 sensor from Empatica was used to collect physiological data. The E4 is a research grade wearable wristband that collects data from a photoplethysmography (PPG) sensor, an electrodermal activity (EDA), an infrared thermopile, and a 3-axis accelerometer. From the PPG, the E4 outputs heart rate data, blood volume pulse, and heart rate variability. The raw EDA signals can be decomposed into relevant features such as skin conductance response and skin conductance level. Body temperature is available from the thermopile and accelerometer data in 3 directions are given from the accelerometer. All data are saved in individual csv files.

In the context of this work, the goal was to ensure that physiological data could be successfully collected during the activities with minimal losses of data. Future work will explore the use of physiological data and machine learning to create predictive models for stress and cognitive load.

# Menu system

In order to make the SAR-VR system acceptable to the LTC staff, it must be easy to operate. Hence, we have designed a graphical user interface for the LTC staff. When the system is launched, the LTC staff member is presented with a simple multi-page menu system. On the first page, the names of the two participants are entered to allow the robot or avatar to provide appropriate feedback directly to each participant. The staff member chooses between the humanoid or animal SAR-based activity. If the humanoid SAR activity is chosen, the IP address of the robot will be requested to ensure that the system is communicating with the correct robot. Finally, a specific SAR humanoid activity, such as Music, is chosen with a designated difficulty level (Level 1, Level 2, or Level 3). At that point the activity begins. If the animal robot is chosen, no IP address is requested as the communication to the Aibo robot is handled through an API key.

The menu system was developed to decrease the number of manual steps, limit human burden, and reduce errors. For example, the wands communicate with the computer using communication ports similar to other peripheral devices. Instead of requesting the LTC staff member to search through multiple communication ports connected to the computer and manually inputting into the menu system, the menu system automatically selects the correct port by comparing the output of the ports to the expected output of the wand. Fig. 4 shows example pages from the menu system.


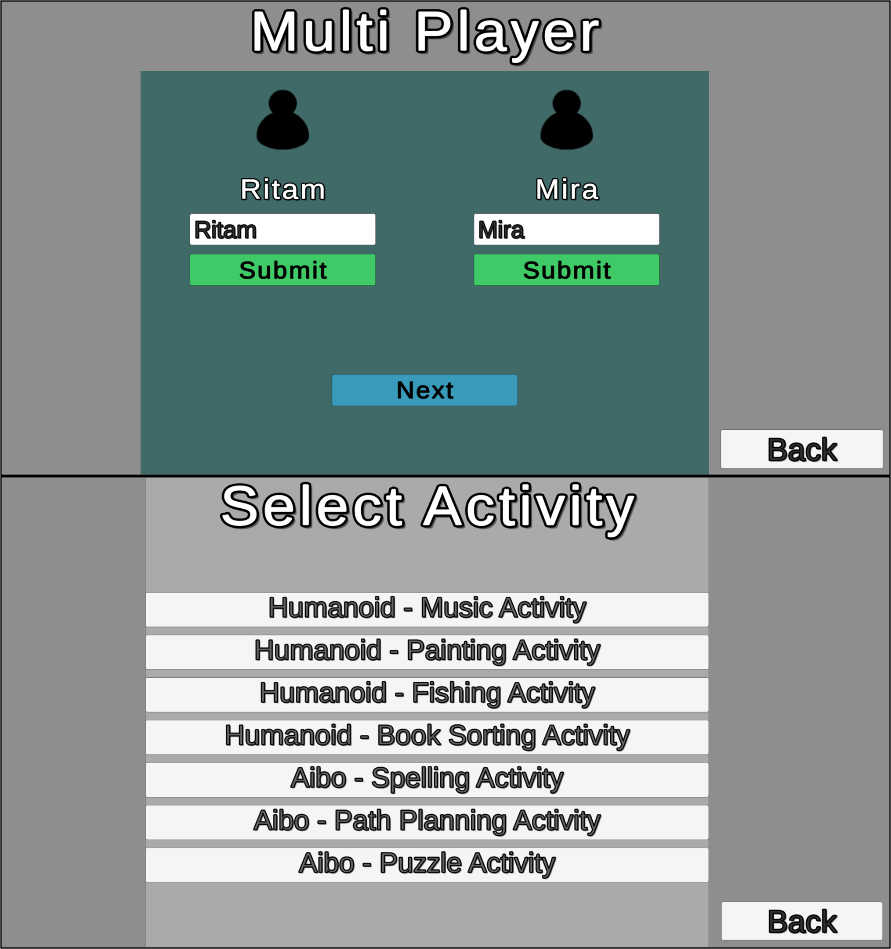


Figure: Top – Player selection screen, bottom – Activity selection screen
