# Supplementary material for: Engaging Older Adults and Staff in the Co-Design and Evaluation of Socially Assistive Robot and Virtual Reality Activities for Long-Term Care: User-Centered Study
Source: JMIR Aging. 2025 Dec 2;8:e75288. doi: 10.2196/75288 (PMC12709162; doi:10.2196/75288)
Supplement: Multimedia Appendix 2 [file aging_v8i1e75288_app2.docx]

Multimedia Appendix 2

**Engaging Older Adults and Staff in the Co-design and Evaluation of Socially Assistive Robot and Virtual Reality Activities for Long-Term Care: A User-Centered Study**

**Description of SAR-VR Activities**

# Activity design

We explored potential virtual activities with the LTC activity directors and older adults residing in LTCs, guided by the framework of multidomain activities that incorporate physical, cognitive, and social stimuli. Based on the conversations that took place during the brainstorming sessions, inspiration for activities was taken from real-life activities that the older adults perform at the LTCs and hobbies they used to enjoy in their youth. Among the potential activities discussed, we began with four initial activities:

1. A *music activity* was designed that requires the participants to play virtual drums along with music from their youth.
2. A *fishing activity* was designed where they can catch virtual fish in a virtual lake environment.
3. A *painting activity* was designed that allowed the participants to paint pictures by numbers.
4. A *spelling activity* was designed that allowed the participants to train Aibo by spelling out dog commands by selecting virtual letters.

The summary of the difficulty levels of each activity can be seen in Table 1.

## Music activity

The music activity utilizes gross motor movement by asking users to perform a drumming motion with the wand to play virtual drums in sync with displayed music notes. It involves cognitive skills of perception and attentional control. It encourages HHI by performing with another person. A menu of songs was provided based on suggestions from the LTC staff and the older adults and included various genres, such as swing, hymns, country, and rock-n-roll.

A software-based audio spectrum analyzer isolates the drumbeats from the music, and musical note symbols are generated in synchronization with these beats. These notes travel down the screen along two vertical bars, the left bar for the left participant and the right bar for the right participant, as can be seen in the Figure. The participants earn points by hitting the drum when the note is crossing a green zone in the bar. Both participants have to perform well to maximize points, which incentivizes participants to encourage one another. In addition to the green zone, higher levels of this activity have a yellow zone above and a red zone below the green zone that indicates the note being played too early or too late, respectively. These extra zones introduced an added layer of cognitive difficulty. NAO guides the participants through the tutorial and provides encouragement and corrective feedback during the activity to increase engagement.

## Fishing activity

The fishing activity utilizes both gross and fine motor skills, as well as problem solving, attention, and working memory. Furthermore, in order to catch the fish and successfully transfer the fish to the bucket, the two participants must coordinate and cooperate to accomplish their shared goal. In the final version of the fishing activity, the participant on the right side controls the fishing pole. The participant makes a casting motion with the wand, which casts a fishing line onto the screen depicting a virtual world of water with a school of fish. At the end of the line is a cursor controlled by the movement of the wand. Using this cursor, the participant can catch a fish. Once captured, the participant on the left must use their wand to control a net. The two participants must coordinate to transfer the fish from the rod to the net. After the fish is successfully transferred, the left player is able to deposit the fish into a virtual bucket to score points. During the activity, NAO provides reminders for next actions, as needed. The feedback is prompted by the state machine if the older adults take too long to perform their steps. Nao also encourages them to seek help from their partner, if required and provides positive reinforcement throughout the activity.

## Painting activity

The painting activity focuses on fine motor skills to precisely guide a paintbrush to the correct segments in an image, while also employing recognition and attention. To complete the full painting, participants must collaborate.

The activity is structured as a paint-by-numbers format where each participant has a set of assigned numbers and is only able to paint the segments corresponding to their numbers. Each participant has a color palette that contains the colors corresponding to the segments they are painting. Using a virtual paintbrush, each participant can choose a color and then guide the paintbrush to the appropriate segment to fill in the color, as shown in the Figure. If a participant faces difficulty performing the activity, NAO provides helpful reminders about the next steps, as well as encouragement. In more complex levels, each participant has an increased number of segments to paint.

## Spelling activity

The spelling activity focuses on fine motor skills for cursor control and cognitive skills to recall and spell a word presented to the participants. This activity uses the dog robot Aibo to entertain the participants upon activity completion and serve as a reward to keep them engaged.

In the virtual environment, a word that corresponds to a dog command (e.g., sit, shake, dance, etc.) is given to the participants. Using the wand, the participants are instructed to choose letters to spell out the command that was prompted. Half of the letters are randomly colored red; the others are blue. Each participant can only choose one color of letters, creating collaboration within the activity. After each word is spelled out, Aibo performs the corresponding trick as a reward. In this activity, a screen-based avatar, shown in the bottom right of the Figure, gives instructions and feedback instead of Nao to separate the effect of the two types of SAR.


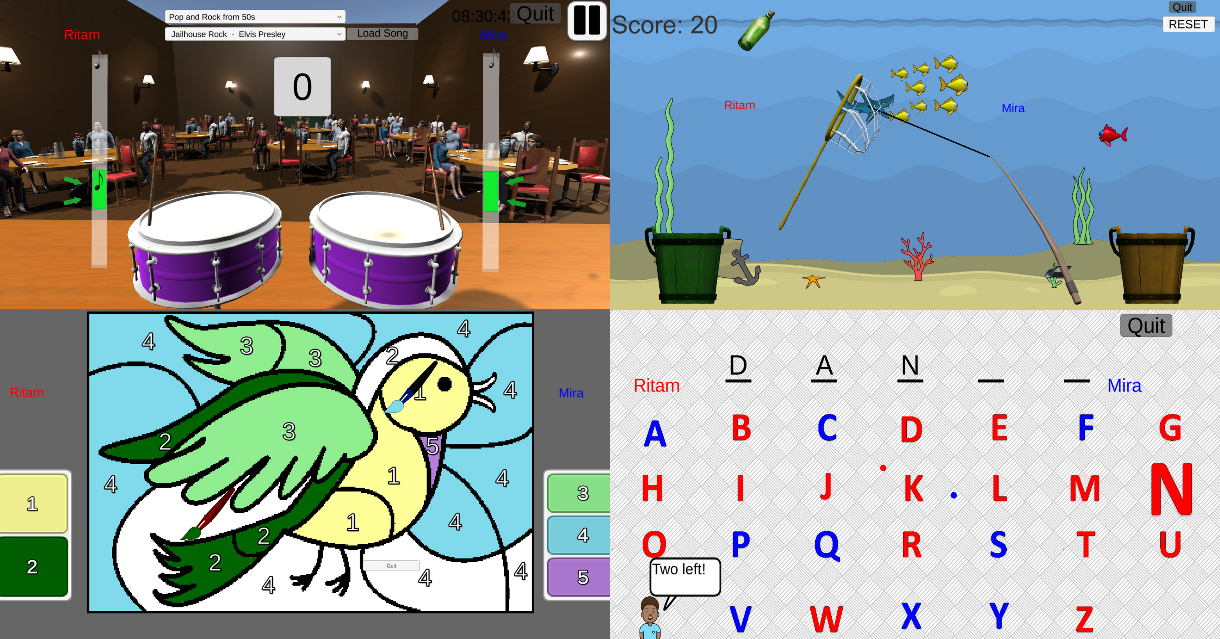


**Table 1. Summary of Difficulty Levels of Activities and Features of Each Level**

| Activity | Difficulty level | Features |
| --- | --- | --- |
| Music | Easy | Free play, participants do not have to follow the notes |
|  | Medium | Participants need to hit the drum when the notes are in the green zone |
|  | Hard | Participants need to hit the drum when the notes are in the green zone but get a warning when they hit it in the yellow or red zones. The additional zones provide greater visual stimuli. |
| Fishing | Easy | All fish and bottles are static |
|  | Medium | The fish and bottle move which requires the participants to track the items |
|  | Hard | The fish and bottles move, and the participants are instructed to deposit the items in a specific basket |
| Painting | Easy | A picture of a rainbow with seven zones to paint |
|  | Medium | A picture of a bird with higher number of zones to paint |
|  | Hard | A picture of an elephant with the highest number of zones to paint |
| Spelling | Easy | Three letter words, letters are static |
|  | Medium | Five letter words, letters bounce around |
|  | Hard | Six letter words, letters move around and need to be tracked |
