# Supplementary material for: Engaging Older Adults and Staff in the Co-Design and Evaluation of Socially Assistive Robot and Virtual Reality Activities for Long-Term Care: User-Centered Study
Source: JMIR Aging. 2025 Dec 2;8:e75288. doi: 10.2196/75288 (PMC12709162; doi:10.2196/75288)
Supplement: Multimedia Appendix 3 [file aging_v8i1e75288_app3.docx]

**Multimedia Appendix 3**

**Engaging Older Adults and Staff in the Co-design and Evaluation of Socially Assistive Robot and Virtual Reality Activities for Long-Term Care: A User-Centered Study**

**Study Set Up Guide**

Setup Guide

This document will contain a full set up guide for all the sensors, hardware, and software we will be using.

Contents

[Room Setup Diagram 2](#_Toc143598011)

[Pre-Session Setup 3](#_Toc143598012)

[Computer Setup 3](#_Toc143598013)

[Alternate Laptop and Lapel Mic Setup 3](#_Toc143598014)

[Router Setup (If Needed) 5](#_Toc143598015)

[Robot Pre-Session Setup 5](#_Toc143598016)

[Nao: 5](#_Toc143598017)

[Aibo: 6](#_Toc143598018)

[E4 Setup 6](#_Toc143598019)

[Video Recorder Setup 6](#_Toc143598020)

[Kinect Setup 7](#_Toc143598021)

[Wand Setup 8](#_Toc143598022)

[Session Setup (When Participants are Present) 9](#_Toc143598023)

[E4 Part 1 9](#_Toc143598024)

[Video Recorder 9](#_Toc143598025)

[Kinect 9](#_Toc143598026)

[E4 Part 2 9](#_Toc143598027)

[Wands 9](#_Toc143598028)

[Socially Assistive Robotics Application 10](#_Toc143598029)

[Steps for the computer: 10](#_Toc143598030)

[If doing a Nao Based Activity (Music, Painting, Fishing, or Book Sorting) 11](#_Toc143598031)

[If doing a Aibo Based Activity (Spelling, Path Planning, Puzzle) 11](#_Toc143598032)

[Activity Specific Information 11](#_Toc143598033)

[Post-Session 12](#_Toc143598034)

[Before the Participants leave the Room 12](#_Toc143598035)

[After the Participants Leave the Room 12](#_Toc143598036)

[Data Uploading 12](#_Toc143598037)

# Room Setup Diagram

This is a suggested setup diagram and will need to be adapted depending on each site space. For example, the Researcher Station may need to be setup on the opposite side of the diagram so that the wires and setup are not blocking the door. Use your best judgment in setting up and use this diagram as reference for where to position things in relation to each other.


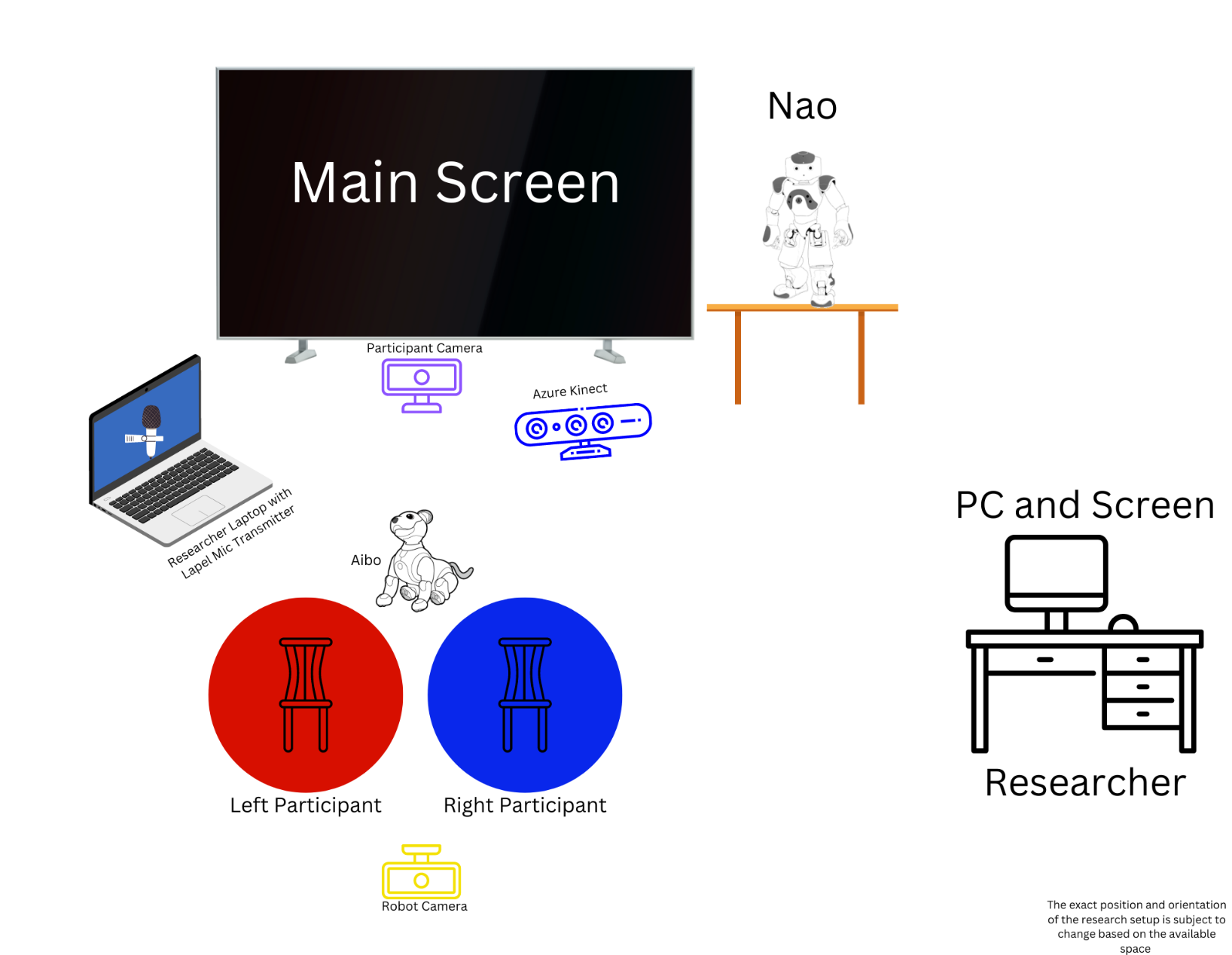


# Pre-Session Setup

## Computer Setup

**Tools needed:**

Computer

Computer power cord

Mouse and Keyboard

Wands

☐ Monitor

☐ Monitor power cord

☐ 3ft Mini Display Port (MDP) to HDMI Cable

☐ 10ft Mini Display Port (MDP) to HDMI Cable

☐ 25ft Power Extension Cable

☐ Power Strip/Surge Protector

**Steps to set up the computer:**

☐ Find the Computer Power Cord (labeled with a red label) and connect it to the computer

☐ Plug the power cable into the surge protector and plug the surge protector into a power socket (use the power extension if needed)

☐ Connect the mouse and keyboard to the computer using the USB cables

☐ Find the Monitor Power Cord (labeled with a red label) and connect it to the monitor

☐ Plug the screen power cord into the surge protector/power strip

☐ Find the 3ft and 10ft MDP to HDMI cables (labeled with orange labels)

☐ Plug the MDP side of the 3ft cable into the computer (labeled with computer side)

☐ Plug the HDMI side of the 3ft cable into the screen (labeled with Screen Side)

☐ Plug the MDP side of the 10ft cable into the computer (labeled with computer side)

☐ Plug the HDMI side of the 10ft cable into the screen being used for the task (projector, tv, etc)

☐ Power on the computer and TV and log in to the computer

☐ this site may need a hotspot depending on if the site’s wifi has firewalls that block the robot's codes. If this is needed, please plug in and power on the hotspot now to allow time for the computer and robot to connect to the hotspot.

During this time, it is a good idea to plug in the wands, E4s and the dog, as they need time to charge prior to the session. Please allot 1 hour in scheduling for set up before the participant comes.

## Alternate Laptop and Lapel Mic Setup

**Tools needed:**

☐ Work Laptop and Laptop Charging Cable

☐ Lapel Mics, Receiver, and Transmitters

☐ AUX to USBC adapter for Dell Latitude 5520 (if needed)

**Steps to set up the Laptop and Lapel Mic:**

☐ Plug in the laptop to the closest outlet on a surface that is close to the participants, Ideally between the participants and the speakers of the TV and NAO

☐ Plug the smartphone adapter (black side) into the left side of the receiver (the one that looks like headphones)

☐ Plug the gray side of the smartphone adapter into the USB C adapter

☐ Plug the USB-c adapter into your computer

☐ Open Sound Settings in Windows and Set the output to your Realtek audio

☐ Set the input to the USB input

☐ Put the microphones on the participants

☐ Record video using camera app on computer

☐ After session, upload video to Sharepoint

## Router Setup (If Needed)

**Tools needed:**

Router

Router power cable

Ethernet Cable

**Steps to set up the router:**

☐ Connect the power cable to the router and the surge protector

☐ Find the Ethernet Cable (Labeled in Orange)

☐ Plug the ethernet cable into the blue internet port on the back of the router

☐ Plug the other side of the ethernet cable^[[1]](#footnote-2)^ into the facilities ethernet port (typically found on the wall)

## Robot Pre-Session Setup

***(only one robot at a time)***

### Nao:

**Tools needed:**

One Nao Robot

**Setting up Nao**

Remove Nao from his box and set him on the table in the squatting position shown below

**
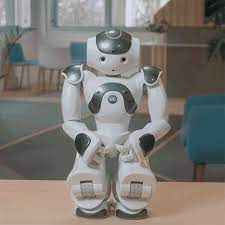

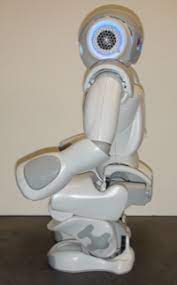
**

Turn on Nao by pressing the button in the center of the robot’s chest **(do not hold the button)**

- - Stay near Nao as he powers up to make sure he is balanced and does not fall
  - Nao will begin to power on, the lights will turn on and it will stand up. Please wait until he is fully standing to proceed
  - Plug in the robots power cable if needed (Nao will tell you out loud if his battery needs charged)

***OR***

### Aibo:

**Tools needed:**

One Aibo Robot

**Setting up Aibo**

Place Aibo on the floor in the position shown below


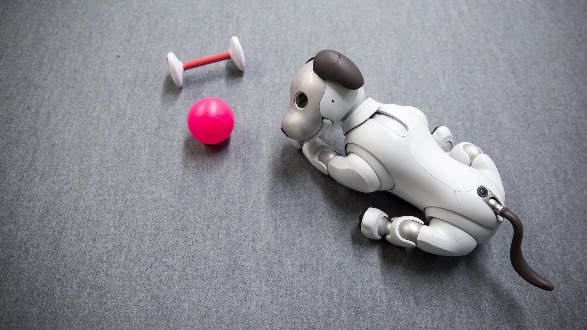


Press and hold the button on his neck until the light turns green

- Aibo will begin to stretch as he wakes up. He is ready to go when he barks twice in a row

## E4 Setup

**Tools needed:**

2 E4 Devices with Chargers

**Prepping the E4:**

Find the E4 Sensors (they are light blue boxes with labels on them). One sensor will be labeled red and the other will be labeled blue.

Wipe down the E4s with disinfectant

You can plug them into the computer to charge while waiting for the participants to arrive.

## Video Recorder Setup

**Tools needed:**

1 webcam labeled as Participant Camera with a Purple Label

1 webcam labeled as Robot Camera with a Yellow Label

1 USB Extension labeled with a Purple Label

1 USB Extension labeled with a Yellow Label

**Setting up the recording system:**

Find the webcams labeled Participant Camera and Robot Camera and mount them both on tripods

Place the camera marked ‘Robot Camera’ behind the two participants facing the robot. Use the diagram as reference

- If the webcam cord is not long enough, use the USB extension cord (labeled with a yellow label)

Place the camera marked ‘Participant Camera’ facing the participants next to the screen. Use the diagram as reference

- If the webcam cord is not long enough, use the USB extension cord (labeled with a purple label)Click here to enter text.

## Kinect Setup

**Tools needed:**

Azure Kinect Sensor

Tripod Mount

Kinect Wires (Kinect power cord and USB cable)

USB Extensions (Data extension labeled with a Blue Label, Power extension labeled with a Red Label)

Computer

Kinect Program

**Setting up the Kinect sensor:**

☐ Mount the Kinect on a Tripod

☐ Set up the Kinect sensor on a stand facing the participants, to the left or right of the monitor or projector, 6 - 7 ft away from the participants

☐ Plug the provided USB cable into the back of the Kinect

- If the USB cord is not long enough to reach the PC, use the USB extension cord (labeled with a blue label)

☐ Use the provided power cable to connect the Kinect to power

- Connect the power USB to the power block and connect it to the surge protector
- If the power cable will not reach the surge protector, use the USB Power extension cord (labeled with a red label). Remove the USB from the Kinect power brick, insert the USB extension, and reconnect the power brick.

## Wand Setup

**Tools needed:**

2 Wands


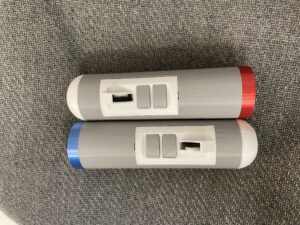


1 Wand Charger


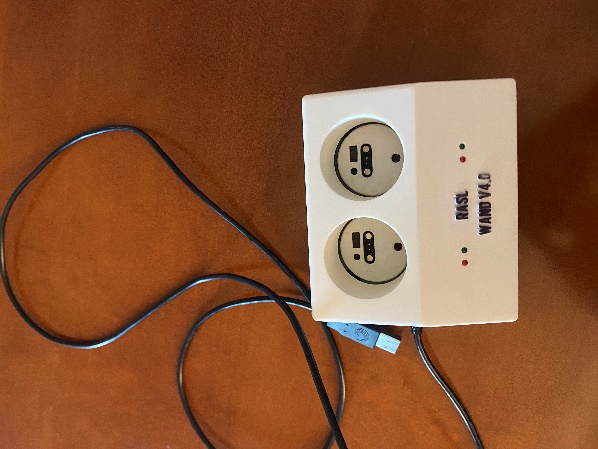


1 Wand Receiver


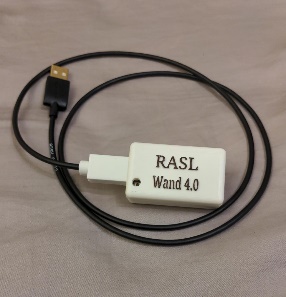


**Prepping the Wands:**

Find the two wands. One wand will have a red bottom and the other will have a blue bottom.

Wipe down the wands with disinfectant

You can plug the charger into the computer and charge the wands while waiting for the participants to arrive.

Plug in wand receiver, this is imperative to wands working, session will not run without this step

# Session Setup (When Participants are Present)

## E4 Part 1

Unplug the E4s and set the chargers aside for the post session steps

Put the E4 with the red label on the non-dominate wrist of the left participant and turn on (red recording light)

Put the E4 with the blue label on the non-dominate wrist of the right participant and turn on (red recording light)

Let the E4 sit on the wrist for up to five minutes before turning on the device to allow the sensors to warm up to body temp

## Video Recorder

Plug both the Robot Camera and the Participant Camera into the PC

Double click on the **OBS** icon on the right side of the Desktop

Make sure there are no old videos in the Video folder of the PC

Press Start Recording

## Kinect

☐ Use the USB cable to connect the Kinect sensor to the computer (USB 3.0 port must be used, those are the blue colored USB ports).

☐ Set up the Kinect sensor on a stand facing the participants, to the left or right of the monitor or projector, 6 - 7 ft away from the participants

On the right side of the computer desktop, double click on the **Kinect** Icon

Press preview button and make sure stick figures of both participants are visible. Adjust the Kinect as needed.

- Press the preview button to make the preview disappear

☐ Press Start Recording

Press the Windows Key + d to return to the desktop

## E4 Part 2

Turn on the E4 by pressing and holding the button for 2 seconds

Wait approximately 1 minute for the light on the E4 to turn red indicating that the device has entered recording mode (the light may turn off to save power, just press the big button one time to see the light again)

Collect at least 3 - 5 minutes of baseline before the task begins

## Wands

Take the Wands off Charge and unplug the Charger if the USB space is needed

Plug in the Wand Receiver into the computer

Turn on the Wands using the switch on the bottom and hand the corresponding wands to each participant

- The left participant will get the Red Wand and the right participant will get the blue wand

Meaning of Colors on Wand

Red: Wand is charging

Red and Green: Wand is almost fully charged but is still charging

Green: Wand is fully charge

## Socially Assistive Robotics Application

### Steps for the computer:

Double click the **Socially Assistive Robotics** icon on the desktop to run the program

When you reach the start menu, choose **Multiplayer**

Enter the participant’s names in order (name of participant sitting on the left facing the screen should go to the left box and the other participant’s name on the right box) and hit submit after each name, press **Next** once the names are submitted

Choose the activity the participants would like to use

Choose the activity level the participants want from dropdown menu

- - Level 1 is the Tutorial
  - Levels 2-4 are the main levels with 2 being the easiest and 4 being the most difficult
    - Find more activity specific information on page 13

DO NOT press Connect or Start. Move onto the robot section below.

### If doing a Nao Based Activity (Music, Painting, Fishing, or Book Sorting)

Get the IP address by pressing the button on the chest once (do not hold, just press)

- -
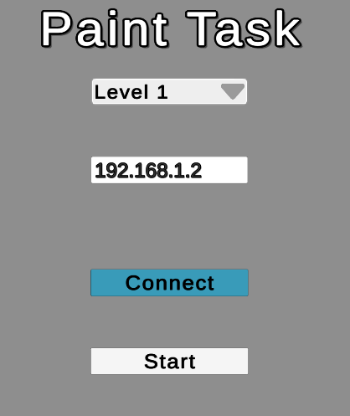
Enter Nao’s IP address in the. Add periods after each number Nao says (e.g., if Nao says ‘192’,’168’,’1’,’2’, the IP address is: 192.168.1.2). The IP address goes in the box immediately below the level selection dropdown. See picture below.

Press Connect on the screen. The Robot will do a squat motion and stand up, once he is done, move to the next step

Make sure the wands are on and the wand dongle is plugged in

Press Start on the screen to being the activity

### If doing a Aibo Based Activity (Spelling, Path Planning, Puzzle)

If Aibo is on and ready, place him on the floor in front of the participants.

Make sure the wands are on and the wand dongle is plugged in

Press Connect on the screen.

Press Start on the screen to being the activity

## Activity Specific Information

**Music Task:**

- - Level 2 is a free play. No notes will appear, the participants can just play the drum as they want

**Spelling Task:**

- - The Dog task has a slider that controls how many additional letters show up. If you move the slider all the way to the right, only the letters that are need for the word will appear.

# Post-Session

### Before the Participants leave the Room

Remove the E4s from the participants and turn them off by pressing and holding the button

Thank the participants for their time and ensure that they receive their gift cards

### After the Participants Leave the Room

#### Socially Assistive Robotics Application

If the application is not already closed, hit quit and then close out of the application.

#### Video Recorder

Press Stop Recording

Close OBS

#### Kinect

Press Save

Once the data is saved successfully, close the application window

***Once there is a final “recordings saved” textbox you can unplug all cameras to plug in E4s***

### Data Uploading

Connect the E4s to the computer using the dock and cord provided in the box

Open the **E4 Manager** on the desktop and press sync sessions for both devices

After syncing, press view sessions

Press the little box with arrow on right side of the screen to open E4 connect and access the data, click on **Sessions**.

Download the two session zip files (match the timestamps and device ids)

Click the **DataManager** icon on the desktop

Once you enter the full facility id (has to match on Sharepoint data storage folder name (i.e. 003)) and the IDs of both participants, a file will be created on the desktop with the facility name, participants IDs, and the current date

Select what you want to happen with the data from the options:

Upload to SharePoint (will require username and password)

Upload to secure drive

If nothing is selected, the zip file will be created on the Desktop, no upload

Press the continue button

☐ ALSO after session, upload extra videos video to Sharepoint

Do not exit out of either page until the file has been fully uploaded

☐ At this time on the laptop search” camera roll” to open your camera roll folder and delete any videos from the session as well as deleting them from your laptop. These videos should not leave the site once they are recorded and uploaded

1. You may need to talk to the facility in order to activate the ethernet port in the testing room. Please plan to have at least one setup day in each facility prior to beginning testing with residents The system will not work without the use of an active ethernet port. [↑](#footnote-ref-2)
