# Supplementary material for: Engaging Older Adults and Staff in the Co-Design and Evaluation of Socially Assistive Robot and Virtual Reality Activities for Long-Term Care: User-Centered Study
Source: JMIR Aging. 2025 Dec 2;8:e75288. doi: 10.2196/75288 (PMC12709162; doi:10.2196/75288)
Supplement: Multimedia Appendix 4 [file aging_v8i1e75288_app4.docx]

Multimedia Appendix 4

**Engaging Older Adults and Staff in the Co-design and Evaluation of Socially Assistive Robot and Virtual Reality Activities for Long-Term Care: A User-Centered Study**

**Questionnaires and Results**

Questions to be asked after **each session activity:**

**Open-Ended Questions:**

1. What did you enjoy about this activity?
2. Is there anything you think we should add or remove from the activity?
3. Would you like to do this activity again in the future?
4. Did you understand the activity? Did you need more instructions?
5. How could we make the activity easier?
6. How could we make the activity harder?

Questions to be asked at the **end of each visit:**


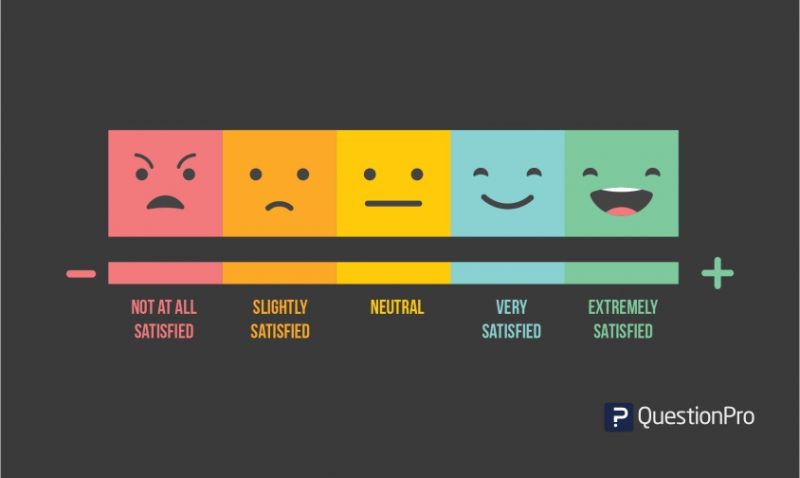


**Using the chart above,**

1. How would you rate your *comfort* level with the wand?
   1. Example answer: I was not at all/extremely comfortable with the wand.
2. How would you rate your *confidence* level with the wand?
   1. Example answer: I was neutral confident with the wand.
3. How would you rate your *comfort* level interacting with the robot?
4. How would you rate your *confidence* level interacting with the robot?
5. How would you rate your *comfort* level interacting with the screen?
6. How would you rate your *confidence* level interacting with the screen?

**Open ended Questions:**

1. What suggestions do you have for the wand?
2. What suggestion do you have for the robot?
3. What suggestions do you have for the screen?


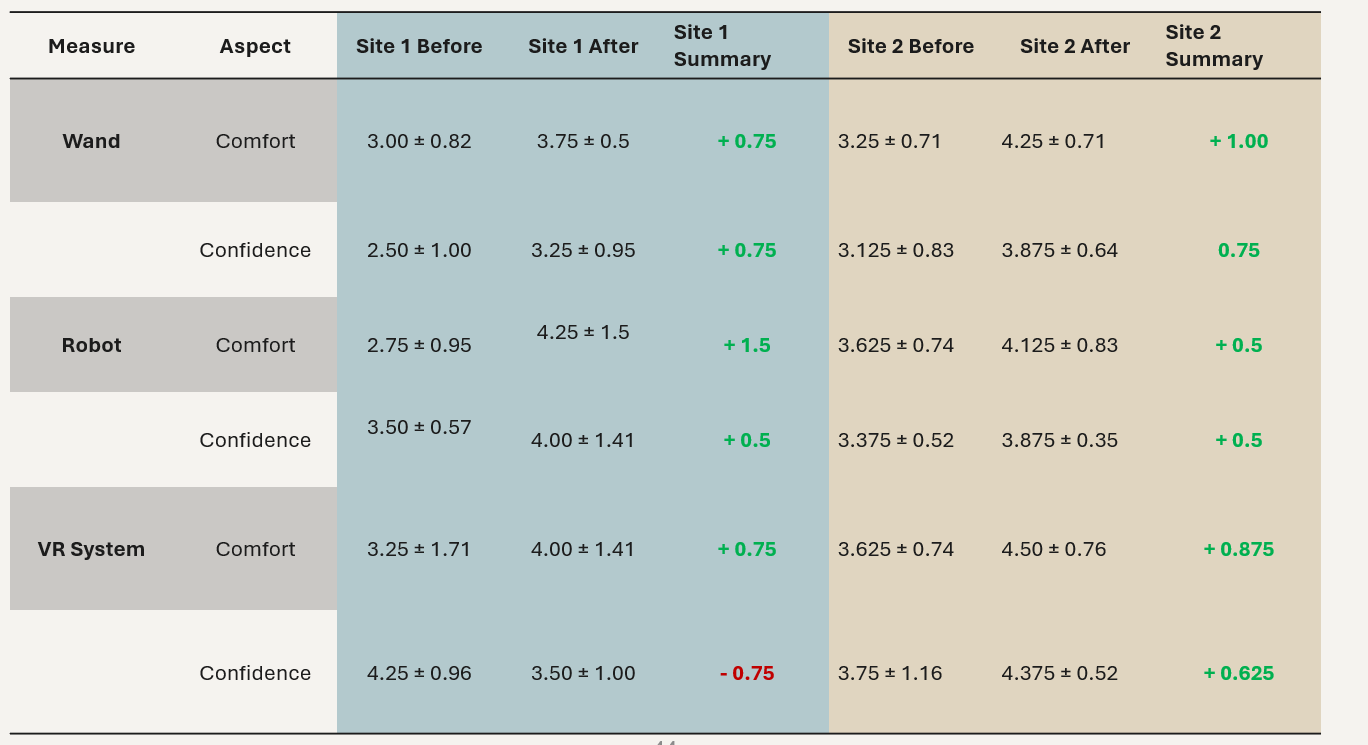


Results of Comfort and Confidence Ratings
